# A Water Droplet Pinning and Heat Transfer Characteristics on an Inclined Hydrophobic Surface

**DOI:** 10.1038/s41598-018-21511-w

**Published:** 2018-02-15

**Authors:** Abdullah Al-Sharafi, Bekir Sami Yilbas, Haider Ali, N. AlAqeeli

**Affiliations:** 1 Mechanical Engineering Department and Centre of Excellence in Renewable Energy, King Fahd University of Petroleum & Minerals, Dhahran, Saudi Arabia; 2 Center of Research Excellence in Renewable Energy (CoRE-RE), King Fahd University of Petroleum and Minerals (KFUPM), Dhahran, 31261 Saudi Arabia

## Abstract

A water droplet pinning on inclined hydrophobic surface is considered and the droplet heat transfer characteristics are examined. Solution crystallization of polycarbonate is carried out to create hydrophobic characteristics on the surface. The pinning state of the water droplet on the extreme inclined hydrophobic surface (0° ≤ δ ≤ 180°, δ being the inclination angle) is assessed. Heat transfer from inclined hydrophobic surface to droplet is simulated for various droplet volumes and inclination angles in line with the experimental conditions. The findings revealed that the hydrophobic surface give rise to large amount of air being trapped within texture, which generates Magdeburg like forces between the droplet meniscus and the textured surface while contributing to droplet pinning at extreme inclination angles. Two counter rotating cells are developed for inclination angle in the range of 0° < δ < 20° and 135° < δ < 180°; however, a single circulation cell is formed inside the droplet for inclination angle of 25° ≤ δ ≤ 135°. The Nusselt number remains high for the range of inclination angle of 45° ≤ δ ≤ 135°. Convection and conduction heat transfer enhances when a single and large circulation cell is formed inside the droplet.

## Introduction

Water droplet on inclined hydrophobic surfaces gives rise to different dynamic behavior when subjected to a gravitational force at different angle of inclination. Depending on the magnitude of adhesion, friction, and shear forces, rolling/sliding or pinning of droplet takes place on the surface. The main forces governing the droplet dynamics in terms of rolling and sliding are the droplet weight and adhesion force generated on the hydrophobic surface due to large droplet contact angle hysteresis. Localized heating of the droplet generates the Marangoni and the buoyancy currents in the droplet fluid while contributing to the droplet dynamics on the hydrophobic surface^1^. The droplet contact angle and the contact angle hysteresis depend on the texture parameters and the free energy of the solid surface^2^. In this case, two possible states can be generated at the surface, which include Wenzel and Cassie and Baxter states^3,4^. On the other hand, the adhesion force resulted between the droplet and the solid surface is mainly governed by the droplet contact angle hysteresis, the droplet size, and the surface tension of the droplet fluid. Increasing the contact angle hysteresis enhances the adhesion force, which is more pronounced for high surface tension fluids and large droplet diameters. Increasing adhesion force at the interface of the droplet and the solid surface modifies the droplet dynamics significantly. In the case of large adhesion forces, the droplet pins on the hydrophobic surface despite the hydrophobic surface undergoes large inclinations. Although surface inclination alters the line of action and the magnitude of the static force in a sessile droplet, the droplet remains on the hydrophobic surface and the initial shape of the droplet deforms according to the force balance. The geometric deformation of the droplet alters the advancing and receding contact angles while changing the adhesion force generated at the droplet and the solid interface. Since local heating generates fluid motion inside the droplet, it contributes to the static behavior and geometric feature of the sessile droplet. Consequently, investigation of the droplet pinning on the inclined hydrophobic surface under local heating conditions becomes essential.

Considerable research studies were carried out to examine the mechanics and heat transfer characteristics of the droplet on the hydrophobic surfaces. A study on the adhesion energy of the liquid droplets located on a hydrophobic flat surface was carried out by Kim *et al*.^5^. They indicated that the existing adhesion force model could not predict the sliding angle correctly because of consideration of the three phase contact line; however, they introduced a new model incorporating the droplet area in contact with the surface. The shape evolution of a sessile water droplet during freezing incorporating the supercooling effect was examined by Zheng *et al*.^6^. They suggested that the final droplet profile was less dependent on the degree of supercooling because it was less affected by the freezing rate. As the cold plate temperature was reduced, the freezing rate increased and the freezing time decreased. The influence of interfacial heat transfer, surface tension, and contact angle on the formation of plasma-sprayed droplets was studied by Zhang *et al*.^7^. They indicated that the interfacial heat transfer was the key parameter influencing the droplet cooling process and the final splat morphology. Increasing the substrate preheating temperatures delayed solidification and low surface tension promoted liquid projection; however, the contact angle was of less importance in changing the surface morphologies. Heat transfer from the heated superhydrophobic surface to droplet for evaporation was investigated by Hays *et al*.^8^. They demonstrated that for supercritical substrate temperatures, as the cavity fraction increased, nucleate boiling was delayed to higher substrate temperatures and the Leidenfrost point occurred at a lower temperature. Marangoni convection instability in a sessile droplet with low volatility on heated surface was examined by Shi *et al*.^9^. The findings revealed that with increasing contact angle, the critical Marangoni number for the onset of convection instabilities first decreased and then increased. The critical cell number of the steady Benard-Marangoni convection kept on only one for contact angle less than 15° whereas it increased sharply after angle lager than 15°. The study for the wettability and condensation heat transfer of sine-shaped micro-grooved surfaces was carried out by Qi *et al*.^10^. They showed that the net force and the sliding velocity increased as droplets grow, and larger geometrical size was favorable to droplets falling. The velocities of sliding down on horizontal grooved surface were less than that corresponding to the smooth surface; however, the velocities of sliding down on vertical grooved surfaces could remain higher than that on smooth surface. A model study for impact, spreading and freezing of a water droplet on horizontal and inclined superhydrophobic cooled surfaces was introduced by Yao *et al*.^11^. They presented a regime map which depicted the different responses of droplets as a function of normal Weber number and Ohnesorge number. The simulation of droplet detachment from solid walls under the influence of gravity was carried out by Tilehboni *et al*.^12^. They showed that the droplet could detach from wettable wall in low Eotvos numbers (Eo ≤ 6); however, in hydrophobic walls, the droplet detached from the wall for Eotvos number in in the range of 3 ≤ Eo ≤ 48. The influence of gravity on the shape of liquid droplet was studied by Ren *et al*.^13^. They demonstrated that the negligible gravity effect enabled to improve the optical performances of the liquid lens by choosing suitable liquids without importance of the density mismatch.

The water droplet attachment and dynamics on the inclined hydrophobic surface is important in terms of heat transfer during condensation or evaporation^14,15^. Heat transfer in a sessile droplet on a hydrophobic surface was investigated previously^16–18^; however, the main focus was the internal fluidity of the droplet on the horizontal surfaces. In these studies, influence of the gravitational force on the flow field and heat transfer was not incorporated. The gravitational force becomes important for reshaping the sessile droplet on the inclined hydrophobic surfaces. This in turn alters the heat transfer rates and modifies the Marangoni and buoyant forces inside the droplet while altering the Nusselt number for different the inclination angles. Consequently, investigation of the heat transfer characteristics of a droplet, which pins on the inclined hydrophobic surface, becomes necessary. The current study presents the new considerations and covers original approaches for droplet pinning and heat transfer on the inclined hydrophobic surface. In the present study, a water droplet attachment (pinning) on an inclined hydrophobic surface is considered and heat transfer from the inclined hydrophobic surface to the pinned droplet is examined. The droplet shape and the heat transfer rates are evaluated for various inclination angles and the droplet volumes. In the analysis, the droplet volume is varied from 10 μL to 25 μL and the inclination angle is changed from 0° to 180°. Experiment is carried out to monitor the droplet shape on the inclined hydrophobic surface. Temperature and flow field inside the droplet are simulated incorporating the experimental conditions. The flow field inside the sessile droplet on the inclined hydrophobic surface is validated through the high speed camera measurements.

## Mathematical Modelling

Internal fluidity of a sessile droplet is simulated in line with the experimental conditions of the inclined hydrophobic surface. The coupled flow and thermal fields are considered simultaneously in the simulations. The continuity equation for transient flow is:

1 $$\frac{\partial \rho }{\partial t}+\nabla .(\rho V)=0$$

where *ρ* is the water density and *V* is the liquid velocity.

For natural convection, the density variation is mainly caused by the thermal expansion of the fluid and can be expressed from Boussinesq approximation as:

2 $$\rho ={\rho }_{o}[1-\beta (T-{T}_{o})]$$

where *β* is the thermal expansion of the water. The momentum equation can be written as:

3 $$\rho (\frac{\partial V}{\partial t}+V\cdot \nabla V)=-{\rho }_{o}\beta (T-{T}_{O})\overrightarrow{g}-\nabla (p-{p}_{o})+\nabla [\mu (\nabla V+{(\nabla V)}^{T})-\frac{2}{3}\mu (\nabla \cdot V)]$$

where *p* is the pressure, *μ* is the dynamic viscosity of the liquid, g is the gravity and *p*_*o*_ is the hydrostatic pressure corresponding to density *ρ*_*o*_ and temperature *T*_*o*_.

The flow field should satisfy the energy balance according to:

4 $$\rho {C}_{p}\frac{\partial T}{\partial t}+\rho {C}_{p}V\cdot \nabla T=\nabla \cdot (k\nabla T)$$

where *C*_*p*_ is the specific heat capacity and *k* is the thermal conductivity.

### Initial Condition

Initially water at stagnant condition is considered inside the droplet; in which case, the flow velocity is set to zero, the pressure is set to the Laplace pressure, and temperature is set to be uniform, which is same as the ambient temperature (300 K). Hydrophobic surface is considered to be at 308 K in line with the experimental conditions.

### Boundary conditions

**Figure 1:**
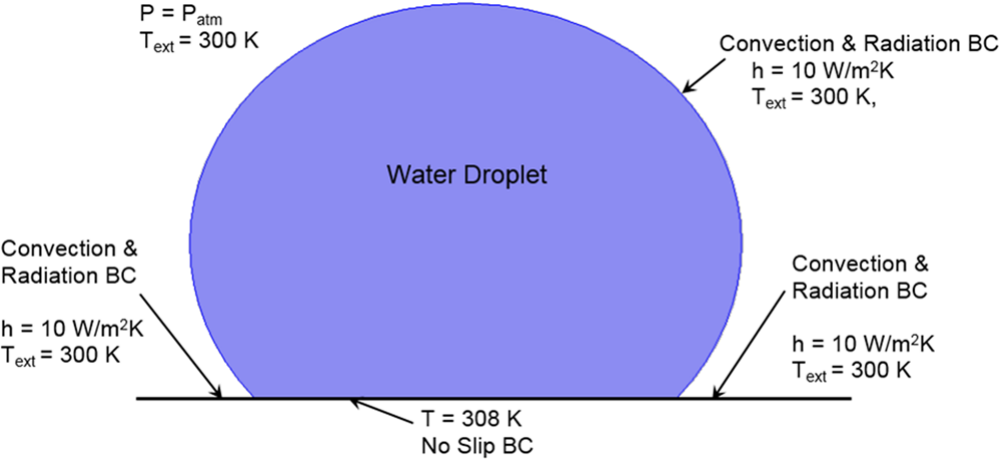
Boundary conditions incorporated in the simulations on the hydrophobic surface.

Figure 1 shows the boundary conditions used in the simulations. The constant pressure boundary is assumed at the droplet outside; in which case, external pressure of the droplet is set at atmospheric pressure. In addition, stagnant air is considered at the droplet outer surface, which yields zero velocity of the air. The natural convection (*h* = 10 W/m^2^K) and the radiation boundary condition is considered at the interface between the droplet free surface and its air ambient, which has temperature of 300 K. Moreover, the natural convection and radiation boundary condition is considered for the free surface of the hydrophobic sample, which is not occupied by the water droplet, i.e., the natural convection and radiation boundary condition is adapted at the hydrophobic surface exposing to the ambient air. Since the hydrophobic surface is maintained at constant temperature, constant temperature boundary condition is adopted at the interface between the droplet bottom and the hydrophobic surface. In addition, no-slip boundary is adopted at droplet and surface interface.

**Figure 2:**
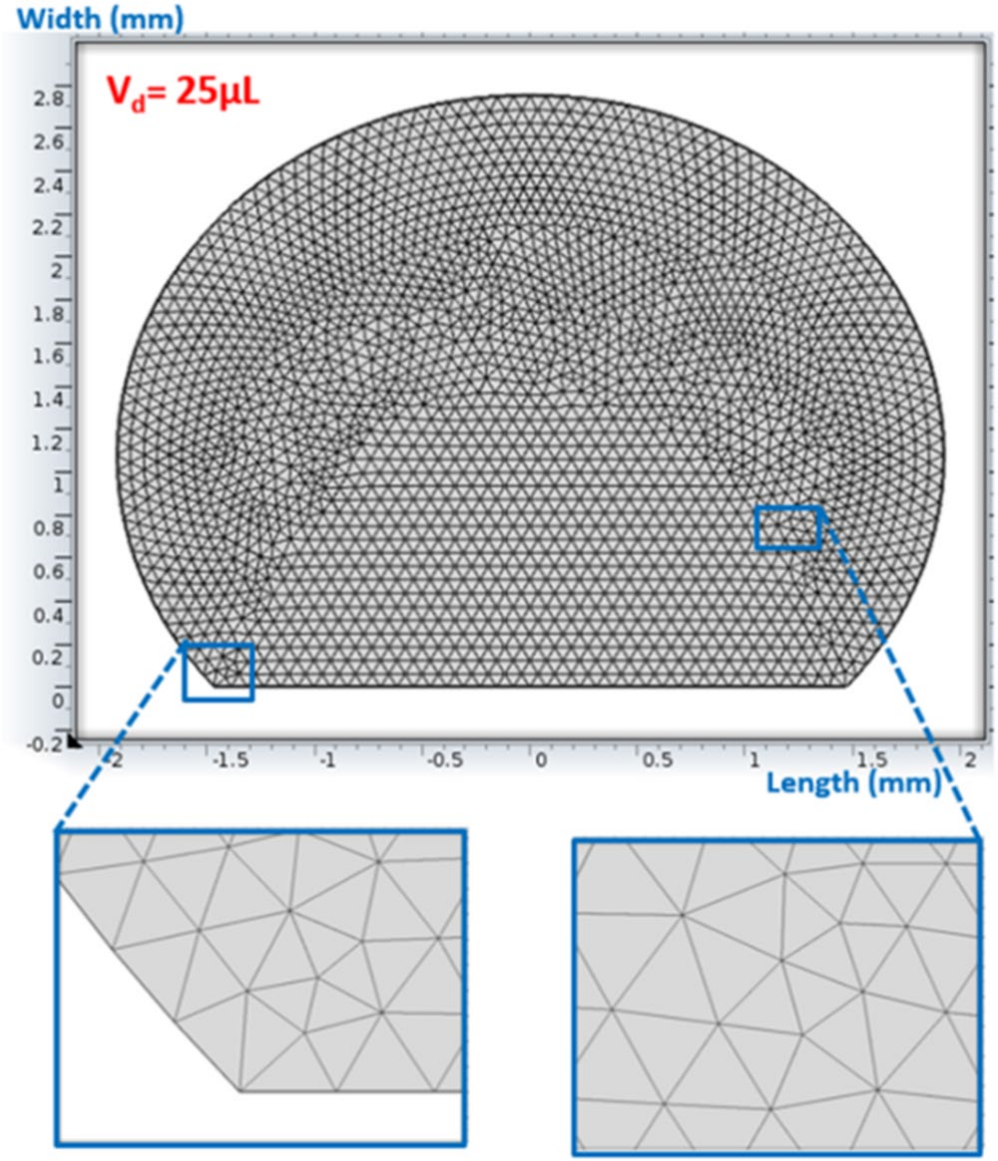
Grid used in the simulations.

**Figure 3:**
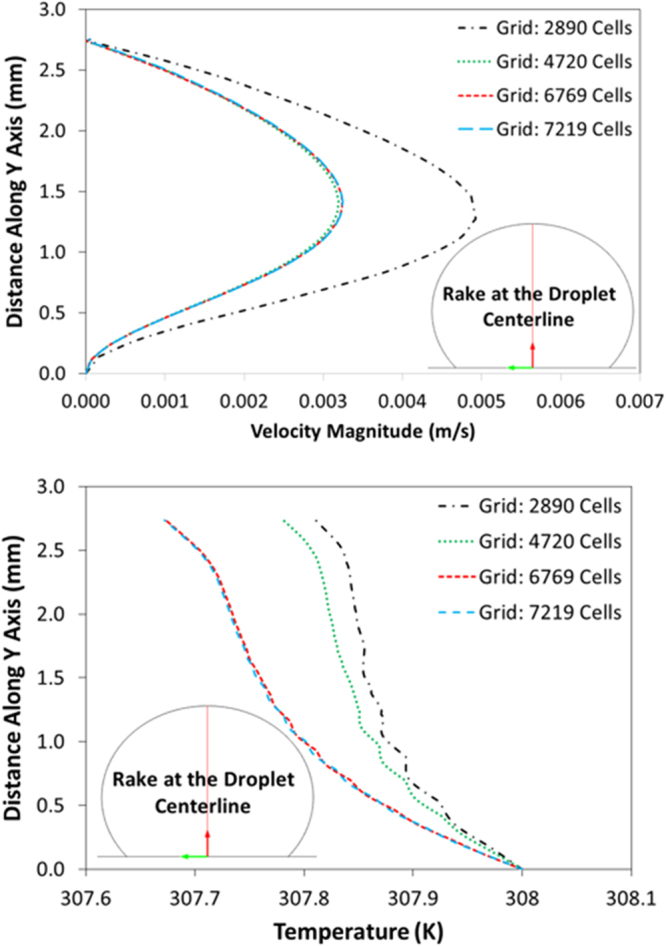
Velocity and temperature distributions along the vertical distance obtained from grid independent tests.

Since the time taken for the experiment and the simulation of experimental conditions is short, which is in the order of 30 s, the evaporation from the droplet surface is neglected in the simulations. This situation is verified during the experiment and droplet images are taken after 30 s and 100 s periods and it is compared with the image corresponding to the droplet at the onset of the experiment. It is noticed that droplet in both images have identical diameters and heights. Therefore, the omission of evaporation from the droplet surface is justified. COMSOL Multiphysics software^19^ is incorporated to simulate the flow field and temperature distribution inside the droplet. Simulations are carried out incorporating 10 μL, 15 μL, 20 μL, and 25 μL water droplet volumes and the inclination angle of the hydrophobic surface are varied within the range of 0° ≤ δ ≤ 180°. Laminar two-phase flow moving mesh model coupled with heat transfer in fluid model is used during the simulations. The models used, solve numerically continuity, momentum and energy equations simultaneously to obtain the flow field and temperature distribution in the solution domain. 3-dimensional simulation of the flow field and temperature distribution is very expensive because of the excessive mesh requirements for the accurate solutions. The comparison of the flow field obtained from 2-dimensional and 3-dimensional simulations are provided in the previous study^16,18^; in which, it was reported 2-dimensional simulations results in very close flow field and temperature distribution inside droplet relative to that obtained from 3-dimensional simulations. Consequently, 2-dimensional simulation of the flow field is adopted in the analysis. In the numerical approach, finer meshes are located in the region where the fluxes are high. Mesh independence tests are conducted for each droplet contact angle considered in the simulations. Figure 2 shows the grid used in the simulations while Fig. 3 shows the grid independent test results for velocity and temperature distributions along the central rake of droplet with 25 μL volume. In this case, after the mesh independence tests, the mesh size comprising of 6769 cells is selected to realize the simulations (for 25 μL droplet). The governing equations of flow are discretized using the backward Euler finite difference method. The implicit scheme with a backward difference approximation is used and unconditionally stable solutions are ensured^20^. The selection of time step is critical to ensure the accuracy of the scheme; in which case, it is in the order of 10^−4^ s. The residuals of flow parameters are set as $|{\psi }^{k}-{\psi }^{k-1}|\le {10}^{-8}$.

## Experimental and Validation of Flow Velocity Inside Droplet

The solution-crystallized polycarbonate surface had hydrophobic characteristics and it was used in the study. The polycarbonate wafers of 3 mm thickness were cleaned prior to immersing into acetone in which the samples were kept for 4 minutes in line with the methods indicated in an earlier study^21^. The hierarchical texture was obtained after solution crystallization of the polycarbonate surface. The crystallized surface composed of micro-/nano-spherules and fibrils. The sessile drop tests were conducted on the crystallized sample surfaces using a goniometer (Kyowa model - DM 501) through which the water droplet contact angle was measured incorporating the technique proposed in the previous studies^22,23^. High-precision drop shape analysis (HPDSA) was incorporated in the measurements^22,23^. The water droplet contact angle is found to be varying from 134° to 136°. A test rig was designed and realized to investigate the droplet pinning on the hydrophobic surface^22^; in which case, the data produced were enabled to analyze the dynamic behavior of the droplets on the inclined hydrophobic surface. To monitor and record the droplet geometric feature on the inclined hydrophobic surface with different angles, Dantec Dynamics (SpeedSense 9040) high-speed camera was utilized. The sample rate was kept at 100 frames per second, which provided image resolution of 960 × 720 pixels.

**Figure 4:**
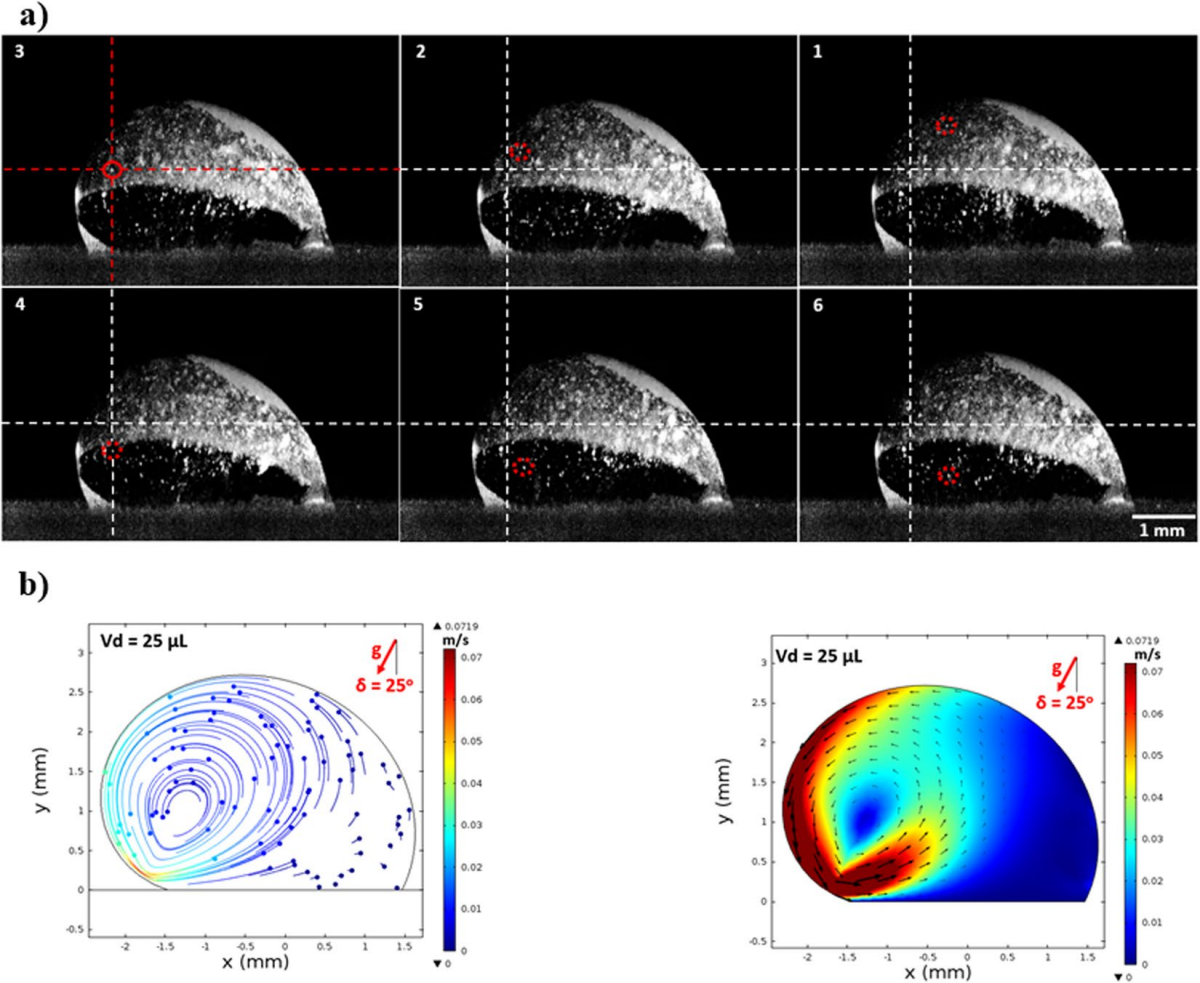
Images obtained from PIV for particles inside droplet during different periods and simulation results: (**a**) Images of particles inside droplet, and (**b**) velocity contours predicted from the simulations in line with experimental conditions.

To validate the flow field inside the droplet, desalinated water was mixed with 3% (by volume) of the hollow glasses with the nominal size of 10 μm. The droplet of this mixture was used to form a droplet located on the 25° inclined solution crystallized surface, which had a constant temperature (308 K). Prior to the measurements, a laser beam plane-illumination was adapted while the beam was passing through the cross-section of the droplet. In the experiments, the droplet volume was selected as 25 μL. The Particle Image Velocimetry (PIV) was used to record the motion of the hollow glass particles across the droplet cross-section. The PIV lens was focused on the droplet cross-section where the laser beam was illuminating and the PIV data were recorded instantly after focusing the the PIV lens. Due to the lens aberration correction of the PIV system, the width of the illuminated plane was slightly larger than the width of the focused plane at the droplet cross-section. However, this difference is considerably small. In addition, simulations were carried out to predict the flow field developed inside the droplet while resembling the water-hollow glass mixture. In this case, the the discrete phase model was used to solve the governing equations of flow while the hollow glass particles were present in the solution domain. Since the hollow glass particles concentration was low in the droplet fluid (3% by volume), a slurry-single fluid model was assumed in the solution of governing equations. Consequently, the effective thermal properties were used in the solution of the equations. The details of the formulation and numerical solution are found in the previous study^24^. In the simulations, the initial and the boundary conditions for momentum and energy equations were set while mimicking the experimental conditions. The trajectory of the hollow glass particles provided tracing of the flow velocities inside the droplet. Table 1 gives the properties of the hollow glass particles. Experiments were repeated several times and the experimental data distribution resulting in the confidence level of 95% was secured. In this case, the mean distribution of the data was within ±1.75 of the standard deviation of the distribution of a single measurement. The experimental uncertainty involved with the experimental measurements was in the order of 4%. The flow velocities predicted and measured from the PIV data are given in Table 2 while Fig. 4 shows the PIV images and the relevant velocity contours predicted from the simulations. It is evident that the data for the velocities are in good agreement; nevertheless, the discrepancies between the findings are small and they can be attributed to the computational errors, such as round-off errors, and the experimental uncertainties.

**Table 1:** Properties of hollow glass spheres at 300 K.

Property of Hollow Glass Particles	Values
Mean particle size (µm)	10
Particle shape	spherical
Density (kg/m^3^)	1400
Melting point (°C)	740
Thermal conductivity (W/mK)	1.14
Specific heat capacity (kJ/kgK)	0.83

**Table 2:** Predicted velocity field and PIV images for hollow glass particles in the droplet.

Particle #	X (mm)	Y (mm)	Simulations V(m/s)	Experiment V(m/s)
1	−1.736133456	0.514478445	0.0132	0.0133
2	−1.723148108	0.761199713	0.0101	0.0100
3	−1.943898797	0.917023659	0.0135	0.0140
4	−1.801060081	1.254642367	0.0091	0.010
5	−1.320602894	1.825996995	0.0073	0.0072
6	−0.93104291	1.890923619	0.0066	0.0066
7	−0.632380247	1.683158278	0.0061	0.0061
8	−0.424614906	1.280612946	0.0072	0.0071
9	−0.37267375	0.930009127	0.0086	0.0082
10	−0.424614906	0.709258437	0.0095	0.0095
11	−0.775218964	0.579405189	0.0132	0.0123
12	−1.086867094	0.410595775	0.0170	0.0165

Flow velocity predicted from simulations and obtained from PIV data. The inclination angle is δ = 25° and droplet volume is 25 μL Please also observe the particle trajectories and relevant simulation results in Fig. (4).

## Results and Discussion

Pinning of water droplet on an inclined hydrophobic surface is considered and the influence of gravitational force on the droplet shape is examined. Heat transfer from inclined hydrophobic surface to water droplet is simulated in line with the experimental conditions. In the simulations, various inclination angles of the hydrophobic surface and four droplet volumes are incorporated.

### Morphology and Hydrophobic Characteristics of Surface

**Figure 5:**
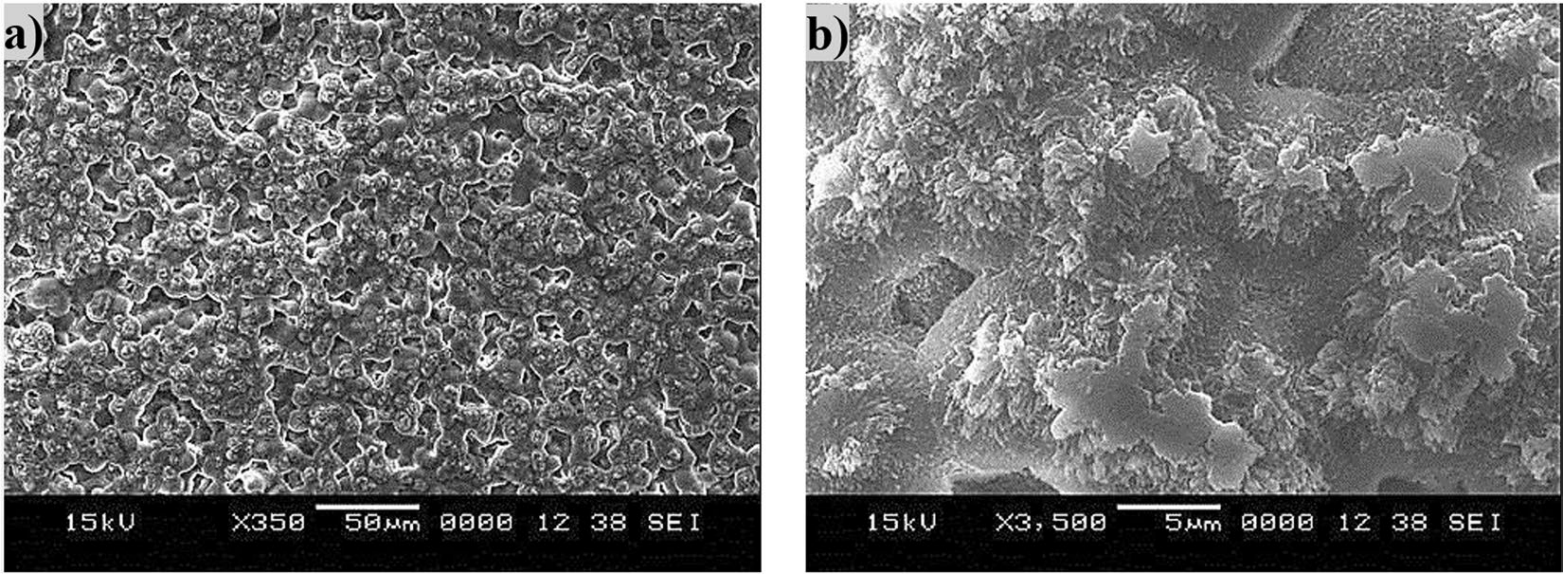
SEM micrograph of solvent crystallized polycarbonate surface: (**a**) texture with spherules, and (**b**) fibrils on spherules surface.

**Figure 6:**
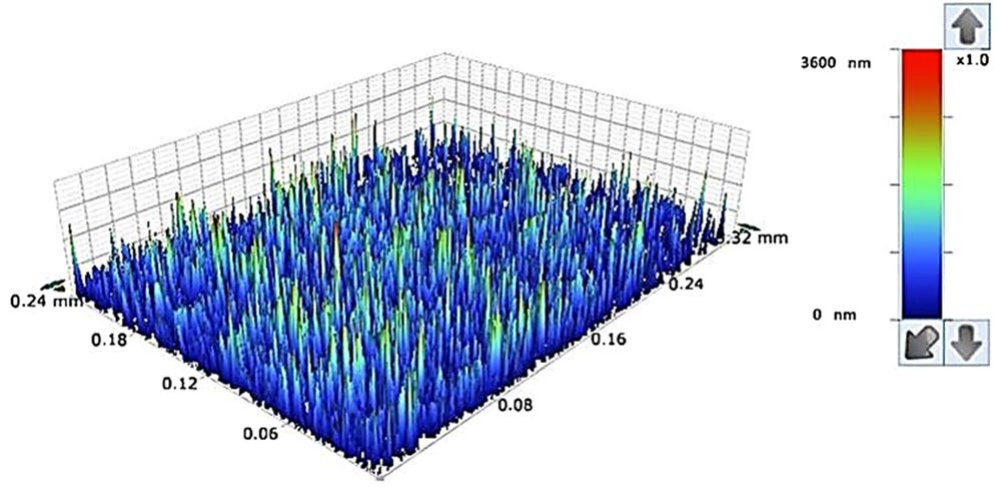
Three-dimensional Optical image of crystallized polycarbonate surface.

**Figure 7:**
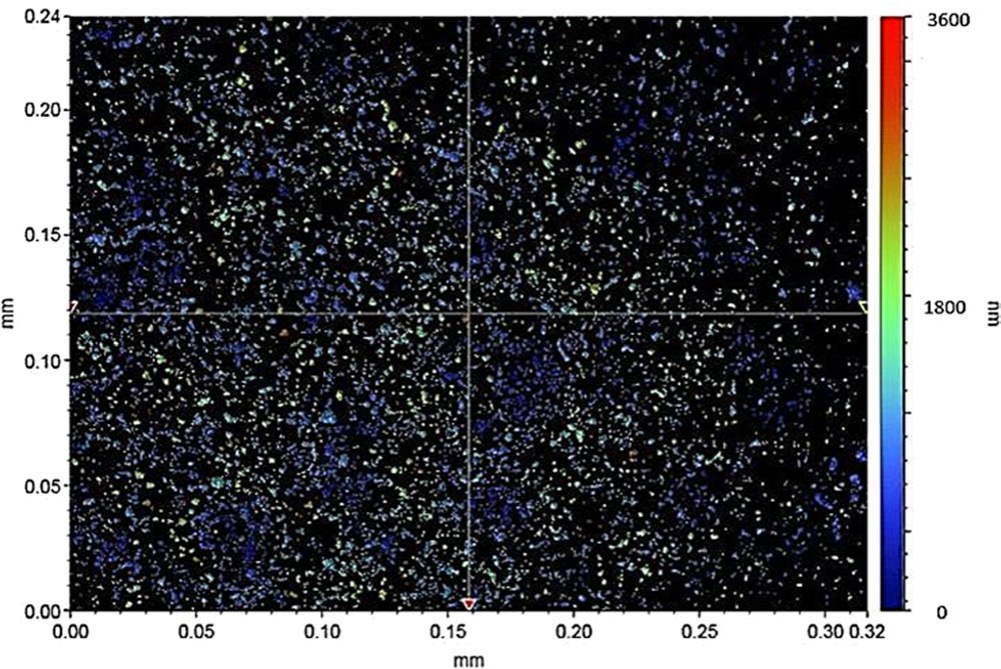
Landscape of texture height for crystallized polycarbonate surface.

**Figure 8:**
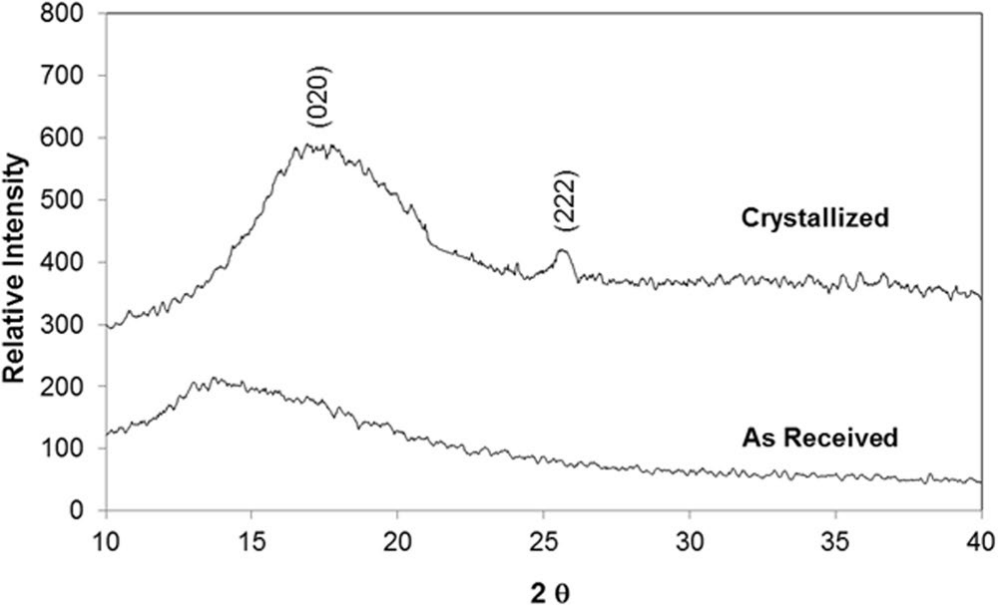
X-ray diffractogram of as received and crystallized polycarbonate surface.

Figure 5 shows SEM micrograph of solvent polycarbonate surface. Crystallization results in hierarchical texture consisting of closely spaced spherules (Fig. 5a) and fibrils (Fig. 5b). The hierarchical texture is also evident from Figs 6 and 7, in which 3-dimensional optical image (Fig. 6) and landscape of surface texture height (Fig. 7) are shown. The size of the spherules is in the order of few micrometers and sub-micrometer size fibrils emanate from the spherules surface. In addition, no asperities such as micro-cracks or large size cavities are observed on the crystalized surfaces. The crystallization takes place in three stages including initiation of crystallization, primary crystallization, and secondary crystallization^25^. During crystallization, a fetus is formed as the polymer chains align in a parallel way and as the process progresses gradually, the chains are added to the fetus. As the fetus size becomes large enough, the growth of nucleus initiates spontaneously^25^. The nucleation resulted in the bundle-like or lamellar crystals formations. The types of crystallization depend on the length of primary nucleus and free energy of the surface normal to the chain direction per unit area^26^. In the case of solution crystallization, the mixture of bundle-like and lamellar nucleus can be formed through the build up by a series of additions of the repeating units during the solution crystallization process. Figure 8 shows X-ray diffractogram of crystallized and as received polycarbonate samples. Since no clearly identifiable peak is observed in the diffractogram of the as received sample, it demonstrates totally amorphous property. In the case of crystallized surface, two peaks are evident in the diffractogram. The locations of these peaks occur at the diffraction angle 17.1° (020 phase) and 25.7° (222 phase). The heights and full width at half maximum (FWHM) of the peak differ from each other. The crystallinity of the solution crystallization can be determined from the ratio of the sum of integrated X-ray peak intensities of the reflections from the crystalline phases to the total scattered X-ray intensity after background subtraction^27^. This arrangement results the crystallinity (*f*_*c*_) values in the order of 13.2%, which is similar that reported in the previous study (13.6%)^27^.

On the other hand, the solvent crystalized surface possesses hierarchical texture with micro/nano size spherules and fibrils and the wetting of the surface is governed by the Cassie and Baxter state. In this case, the resulting surface texture gives rise to the air pockets, which are trapped in the texture while resulting in Cassie-Baxter state on the crystallized polycarbonate surface. The apparent contact angle including surface roughness can provide more realistic formulation of the liquid droplet contact angle^28^. A liquid droplet has liquid-solid and liquid-vapor interfaces and the apparent contact angle equation incorporates the contributions of these interfaces. Hence, the equation for the apparent contact angle becomes^28^:

5 $$cos{\theta }_{c}={f}_{1}cos{\theta }_{1}+{f}_{2}cos{\theta }_{2}$$

where *θ*_*c*_ is the apparent contact angle, *f*_1_ is the surface fraction of liquid-solid interface, *f*_2_ is the surface fraction of liquid-vapor interface, *θ*_1_ is the contact angle for liquid-solid interface, and *θ*_2_ is the contact angle for liquid-vapor interface. For the air-liquid interface, *f*_1_ can be represented as *f*, which is the solid fraction, and air fraction (*f*_2_) becomes (1 − *f*). The parameter *f* ranges from 0 to 1; in which case, *f* = 0 is the case where the liquid droplet is not in contact with the surface and *f* = 1 is case where the surface is completely wetted. However, the contact mode changes from Cassie-Baxter state to Wenzel state^29^ when the surface texture becomes sparse or when the droplets impact the surface with high velocity^30^. The solid fraction of the wetted surface area (*f*) can be associated with the surface roughness ratio (*r*). Therefore, the solid fraction of the surface can be formulated from the projected area of spherules over the total projected area of the textured surface. This corresponds to the fraction of the area covered by the spherules at the crystallized surface. The fraction of the area wetted by the liquid can be written as,

6 $$f=\frac{(Projected\,Total\,Textured\,Area)-(Area\,of\,Liquid\_Air\,Interface)}{(Projected\,Total\,Textured\,Area)}$$

**Figure 9:**
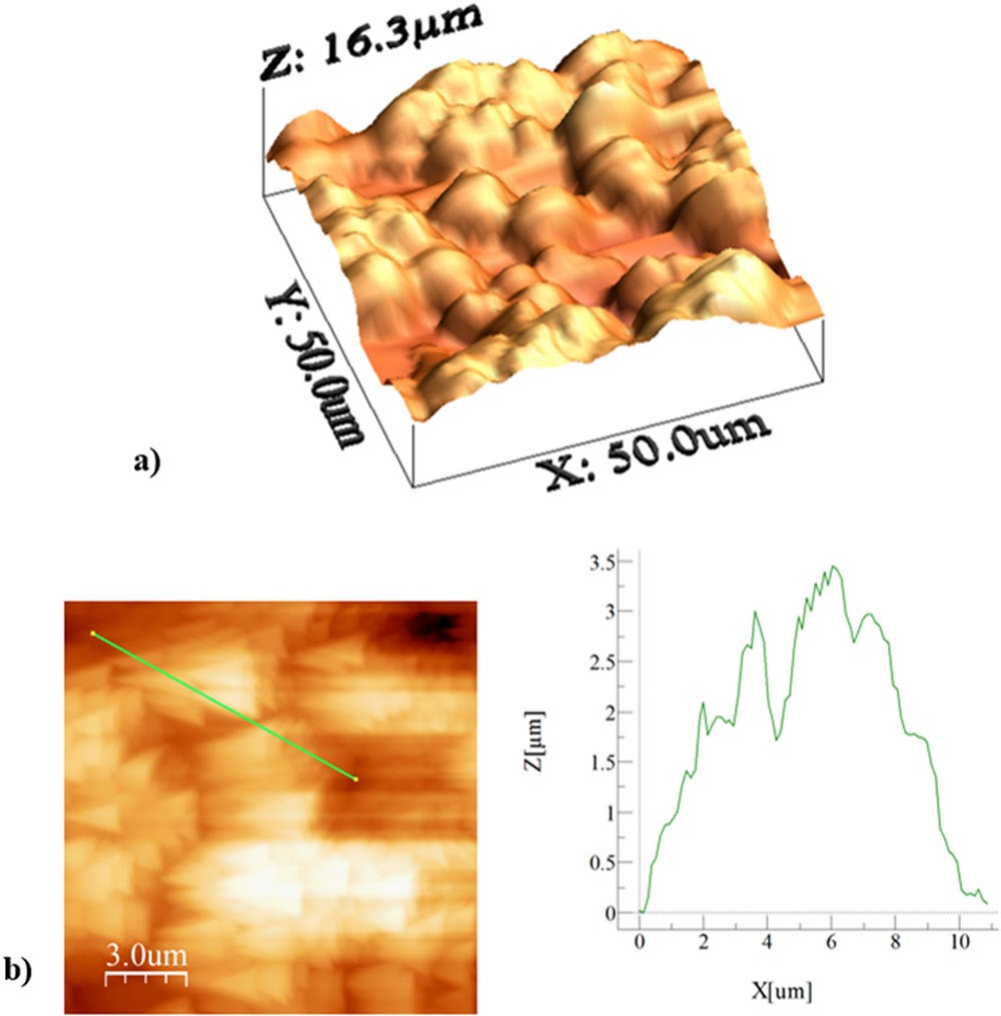
AFM images and line scan of crystallized polycarbonate surface: (**a**) 3-dimensional image of surface, and (**b**) line scan on textured surface.

The data related to the areas of the textured and the air liquid interfaces are determined from AFM image. Figure 9a shows AFM image of three-dimensional crystallized surface while Fig. 9b shows the AFM line scan of the crystallized surface. The presence of spherules and fibrils is evident on the crystallized surface. The spherules form some closed packed of texture gaps, which are filled by the air captured below the droplet meniscus on the surface. Some small oscillations on the peaks of spherules represent the sub-micron size fibrils. This situation can be observed from the surface line scan (Fig. 9b). The average surface roughness is in the order of 3.4 μm. The solid fraction for the solvent induced crystallized polycarbonate surface is determined to be within the range of 0.4 ≤ *f* ≤ 0.6. It should be noted that the solid fraction is determined from 3-D Optical Profilometer data (discretized 3-D image of the crystallized surface) and it corresponds to the projected of area of all spherules over the projected area of the whole surface. The solid fraction obtained from 3-D profilometer is also compared with the data obtained from AFM 3-D image of the surface. Several repeats of 3-D optical imaging are carried out to cover the large area of the surface, which is in the order of 100 mm^2^. The findings reveal that the solid fraction varies with in 0.4 ≤ *f* ≤ 0.6; however, statistically, over 90% of the data remains within the range of 0.6 ≥ *f* ≥ 0.57 and the average value of the solid fraction is in the order of *f* = 0.58. Moreover, several tests are carried out for the water droplet contact angle measurements on the crystallized surface in line with the previous studies^22,23^ to ensure the correct measurement of the droplet contact angle. Table 3 gives the droplet receding and advancing angles, and contact angle hysteresis for various inclination angles. The contact angle hysteresis is determined from *θ*_*hysteresis*_ = *θ*_*A*_ − *θ*_*R*_, where *θ*_*A*_ is the advancing angle and *θ*_*R*_ is the receding angle. The attainment of large values of the contact angle hysteresis indicates the presence of the large adhesion force, which acts along the three phase contact line at the interface of the droplet on the crystallized surface.

**Table 3:** Receding (θ_R_) and advancing (θ_A_) contact angles measurement for crystallized polycarbonate surface for various inclination angles (δ).

δ (Degree)	θ_R_ (Degree)	θ_A_ (Degree)	θ_R_ (Degree)	θ_A_ (Degree)
	**V**_**d**_ ** = 10 μL**		**V**_**d**_ ** = 15 μL**	
0	134.09	134.76	136.41	135.95
30	121.61	146.73	119.29	156.65
60	110.78	155.03	103.75	166.92
90	105.22	152.32	95.25	162.8
120	107.68	146.07	95.33	150.46
150	112.38	132.4	105.64	135.48
180	120.76	122.05	117.11	121.31
	**V**_**d**_ = **20 μL**		**V**_**d**_ = **25 μL**	
0	136.74	138.53	135	135.83
30	109.58	156.01	109.62	159.44
60	92.68	166.76	86.7	169.6
90	82.38	159.6	77.22	166.72
120	86.19	145.14	80.81	149.17
150	93.41	131.8	94.71	131.55
180	109.9	110.34	111.66	108.79

The measurement errors lies in between +2°/−2°.

**Figure 10:**
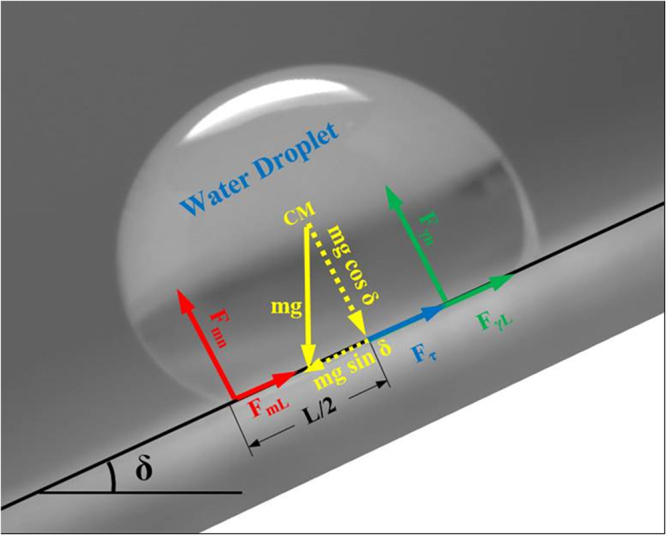
Optical image of the droplet and schematic view of the forces acting on inclined hydrophobic surface.

In general, the buoyant convection can cause the rolling off the droplets from the hydrophobic surface, due to instability, which is true for the droplet volumes *∀* < 0.11 μL^31^. Therefore, the droplet volumes, which are considered in the analysis (10 μL ≤ *∀* ≤ 25 μL) are larger than those reported in the previous study^31^. In addition, the droplet rolling is also not observed during the experiments. On the other hand, the force required to detach a droplet from the surface is same as the force needed to overcome the work of pinning^32^. The adhesion composes of the lateral force (in the plane of the hydrophobic surface) and the normal force (normal to the hydrophobic surface). The normal force becomes important when the hydrophobic surface is extremely inclined (δ ≥ 90°). The pressure of air captured within the surface texture, below the droplet bottom meniscus, remains slightly higher than the atmospheric pressure because of the curvy nature of the droplet meniscus. It should be noted that droplet meniscus forms a curvy arc on the top of the texture. Once the droplet is inclined on the surface, the curvature of the meniscus arc changes while altering the pressure of the trapped air in the texture. Depending on the angle of inclination of the hydrophobic surface, the air volume in the texture can either be increased or decreased slightly. Once the inclination angle increases more than 90°, the air volume in the texture increases while reducing the trapped air pressure and generating Magdeburg like forces. These forces are related to $\sim (1-f){\rm{\Delta }}P{A}_{total}$, where *f* is the solid fraction, Δ*P* is the trapped air pressure change within a single texture gap (cavity), and *A*_*total*_ is the total area of textured surface. The formulation of Magdeburg like forces and trapped air pressure change in the texture gap is given in Supplementary Material S1. These forces contribute to the adhesion of the droplet on the inclined textured surface. However, at some texture locations, pressure may remain as the atmospheric pressure because of the fully connection of the texture gaps to the free atmosphere. In this case, the forces generated within texture gap, due to pressure drop during volume increase of the trapped air within the texture, become zero. On the other hand, the lateral adhesion force is formulated heuristically by approximating the three-phase contact line with a single ellipse and using the experimentally obtained polynomial function for the dependence of the contact angle on the position along the three-phase contact line^33^. Therefore, the resulting equation for adhesion force becomes^33,34^:

7 $${F}_{\gamma L}=\frac{24}{{\pi }^{3}}{\gamma }_{LV}D(cos{\theta }_{R}-cos{\theta }_{A})$$

where *γ*_*LV*_ is the surface tension of the liquid on the solid surface, D is the droplet diameter prior to deformation, *θ*_*R*_ is the receding angle, and *θ*_*A*_ is the advancing angle. The polycarbonate surface is crystallized and it possesses a hierarchical texture; therefore, the roughness parameter can be introduced in Equation 7 pertinent to the Young-Dupre Equation^35^. Consequently, Equation 7 can be written including the roughness parameter, i.e.:

8 $${F}_{\gamma L}=\frac{24}{{\pi }^{3}}{\gamma }_{LV}Df(cos{\theta }_{R}-cos{\theta }_{A})$$

where *f* is the solid surface fraction (solid/liquid contact fraction) as obtained from AFM data, which varies within the range of 0.4 to 0.6. The force diagram resembling the droplet attachment on the surface is shown in Fig. 10. The droplet pinning results in zero net lateral force in the plane of the hydrophobic surface. The net lateral force comprises of the gravitational force component in the plane of the hydrophobic surface, shear force at the wetted surface, the adhesion force, and the Magdeburg like forces. The force balance in the lateral direction of the inclined hydrophobic surface yields:

9 $$mgsin\delta -\{(\frac{24}{\pi }{\gamma }_{LV}D{f}_{1}(cos{\theta }_{R}-cos{\theta }_{A})+{F}_{mL}+{F}_{\tau }\}=0$$

where, *F*_*τ*_ is the shear force generated by the flow field inside the droplet (${F}_{\tau }={A}_{w}\mu \frac{\partial V}{\partial s}$, *A*_*w*_ is the wetted area, *μ* is the fluid viscosity, ∂V/∂s is the rate of fluid strain), which is the same order as the shear force acting on the wetted surface due to flow circulation in the droplet, *F*_*mL*_ is the lateral component of the Magdeburg like force, *m* is the droplet mass, *g* is the gravity, and *δ* is the inclination angle of the surface (Fig. 10). It should be noted that the force generated due to fluid acceleration inside the droplet because of bulging (during inclination of the surface) and heat transfer is assumed to be negligibly small as compared to the gravitational acceleration; therefore, the force acting on the droplet due to gravity and fluid acceleration is considered to be same as the component of the droplet weight in the lateral direction. For the pinning droplet, the normal component of the net force, which is normal to the surface, also remains zero during the inclination of the surface. The total normal force acting on the hydrophobic surface is associated with the normal component of the forces due to tension force, gravity, and Magdeburg like affects. Therefore, the force balance normal to the surface yields:

10 $${F}_{\gamma n}+{F}_{mn}-mgcos\delta =0$$

where ${F}_{\gamma n}$ is the normal component of the surface tension force, and *F*_*mn*_ is the normal component of the Magdeburg like force generated due to trapped air volume expansion within the texture because of the minute change of the droplet meniscus curvature during inclination. Since the surface tension force has two components, namely lateral and normal forces, the normal component can be written as:

11 $${F}_{\gamma n}=\sqrt{{F}_{\gamma }^{2}-{F}_{\gamma L}^{2}}$$

where ${F}_{\gamma n}$ is the normal component of the surface tension force, *F*_*γ*_ is the overall surface tension force, and ${F}_{\gamma L}$ is the lateral component of the surface tension force. The lateral component of the surface tension force is same as that is given in Equation 8. After incorporating the corrected length of the three phase contact line and introducing the solid surface fraction, the surface tension force becomes:

12 $${F}_{\gamma }=\frac{24}{{\pi }^{3}}{\gamma }_{LV}Df$$

**Figure 11:**
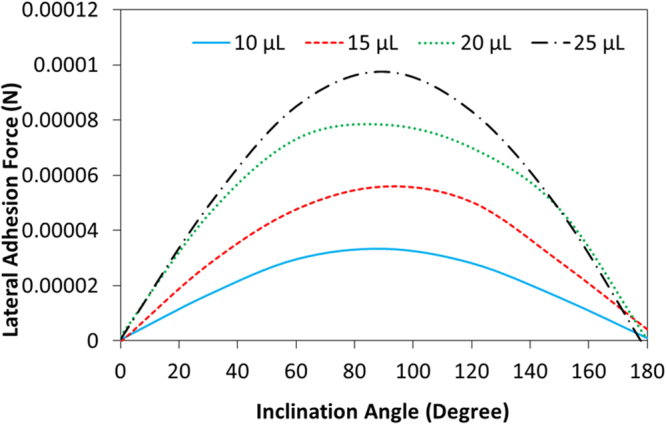
Lateral component of adhesion force with inclination angle for various droplet volumes.

**Figure 12:**
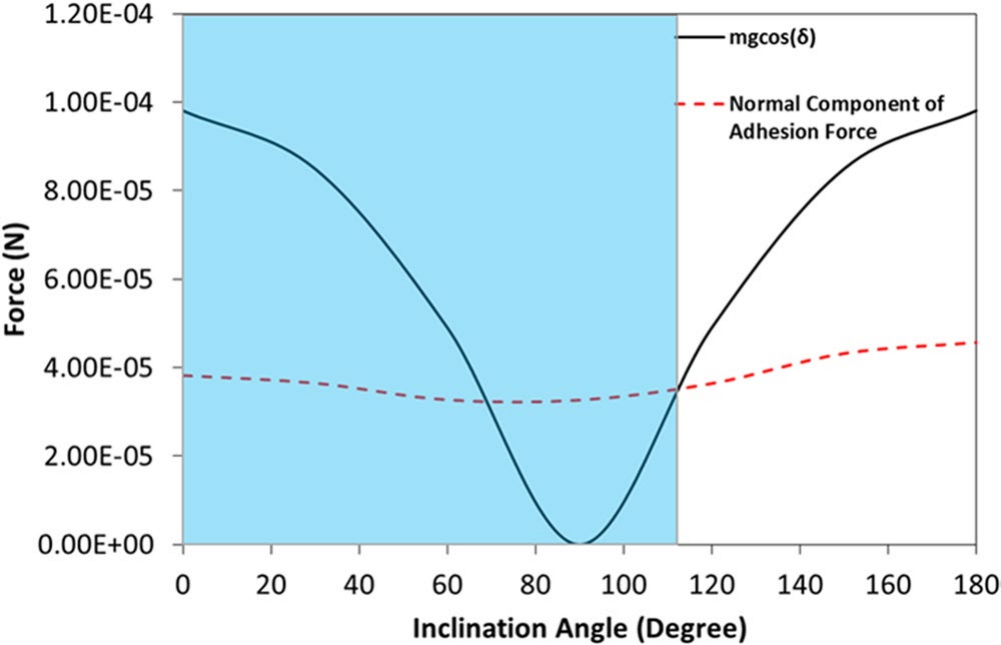
Normal component of adhesion force (*F*_*γn*_) and gravitational force (*mgcos*(*δ*)) normal to hydrophobic surface with inclination angle. The droplet volume is 10 μL.

Figure 11 shows the lateral component of the adhesion force obtained from Equation 8 with various inclination angles and different droplet sizes. It should be noted that the advancing and receding angles of the droplet for a given inclination angle are taken from the experimental data (Table 3). The contact angle hysteresis changes slightly with droplet size because of the increased bulging of the droplet with increasing inclination of the hydrophobic surface (Table 3). The adhesion force determined from Equation 8, firstly, increases with increasing inclination angle, which is more pronounced for the large volume droplets. This behavior is associated with the increased three-phase contact line and changing the receding and advancing angles of the droplet with increasing droplet volume (Table 3) despite the fact that the mass of the droplet increases, which tend to reduce the adhesion force (Equation 9). The lateral adhesion force reaches its maximum at the inclination angle of 90°. In this case, because of the droplet excessive bulging in the direction of gravity, the Magdeburg like forces becomes important for droplet adhesion on the hydrophobic surface. When comparing the lateral adhesion force (Equation 8) and the droplet weight for 10 μL droplet volume and 90° inclination angle, the lateral component of the adhesion force remains less than the droplet weight. However, the shear force (8 × 10^−12^ N), which is generated due to rate of fluid strain in the region of the droplet bottom, is significantly smaller than that of the lateral adhesion force (4 × 10^−5^ N). Therefore, to sustain the droplet pinning at 90°, the force generated becomes higher than the lateral adhesion force. The contribution of Magdeburg like force in the lateral direction is considerable, which is in the order of 10^−4^ N (Equation 9). The variation of the normal component of the surface tension and droplet weight in the normal direction is shown in Fig. 12 with the inclination angle of the hydrophobic surface for 10 μL droplet volume. The normal component of the surface tension force overcomes the normal component of the gravitational force ($mgcos\delta$) in between the inclination angle of 70° ≤ δ ≤ 115°; however, as the inclination angle increases further, the normal component of the gravitational force remains higher than the normal component of the surface tension force. Consequently, the Magdeburg like forces cause droplet pinning on the hydrophobic surface for the inclination angle beyond 115°. It should be noted that the gravitational force is larger than the normal component of the adhesion force for the inclination angle in between 0 ≤ δ < 70°; however, because of the droplet position, which is in the upper surface of the workpiece within the range of these angles, the normal component of the gravitational force does not influence droplet falling from the surface.

**Figure 13:**
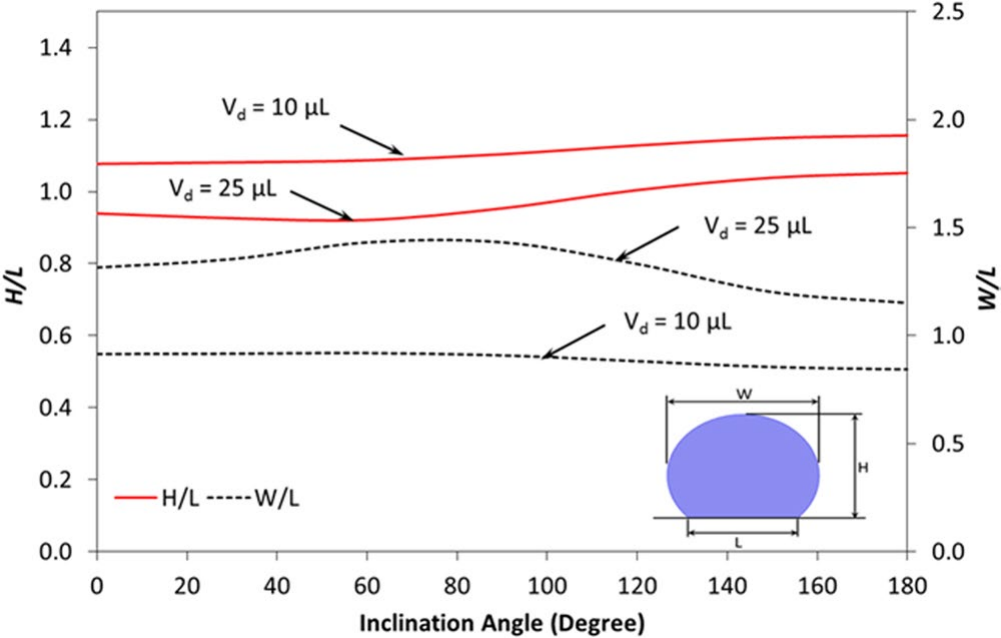
Vertical and lateral deformation parameters of the droplet with the inclination angle for various droplet volumes.

Two deformation parameters are introduced to assess the geometric changes of the droplet due to bulging under the various inclinations of the hydrophobic surface. These include the vertical and lateral deformation parameters. The vertical deformation parameter (*H*/*L*) corresponds to the ratio of the maximum droplet height (*H*) over the wetted length (*L*) of the droplet on the hydrophobic surface while lateral deformation parameter (*W*/*L*) is the ratio of the maximum width (*W*) of the droplet over the wetted length (*L*) of the droplet on the hydrophobic surface. It should be noted that the wetted length of the droplet on the hydrophobic surface changes with the droplet volume; however, it remains constant for a unique droplet volume. Figure 13 shows the vertical and lateral deformation parameters of the droplet with the inclination angle for various droplet volumes. Both deformation parameters increase with the inclination angle. In addition, increasing droplet volume alters both deformation parameters. In this case, vertical parameter remains lower for large volume droplet than that corresponding to the small droplets. On the contrarily, the lateral deformation parameter remains higher for large droplet volume. The behavior of vertical and lateral droplet parameters are associated with the droplet bulging during the inclination; in which case, as the droplet height reduces the droplet width increases, since droplet volume remains constant during the inclination. Moreover, the variation in the vertical and lateral droplet parameters remains small with the inclination angle. This behavior is attributed to the Laplace pressure, which increases with reducing droplet diameter, i.e. droplet tends to behave like a solid marble on the inclined surface as the droplet volume reduces. Consequently, inclination of the surface results in larger variation in the droplet height and width for large volume droplet than that corresponding to the small volume droplet.

**Figure 14:**
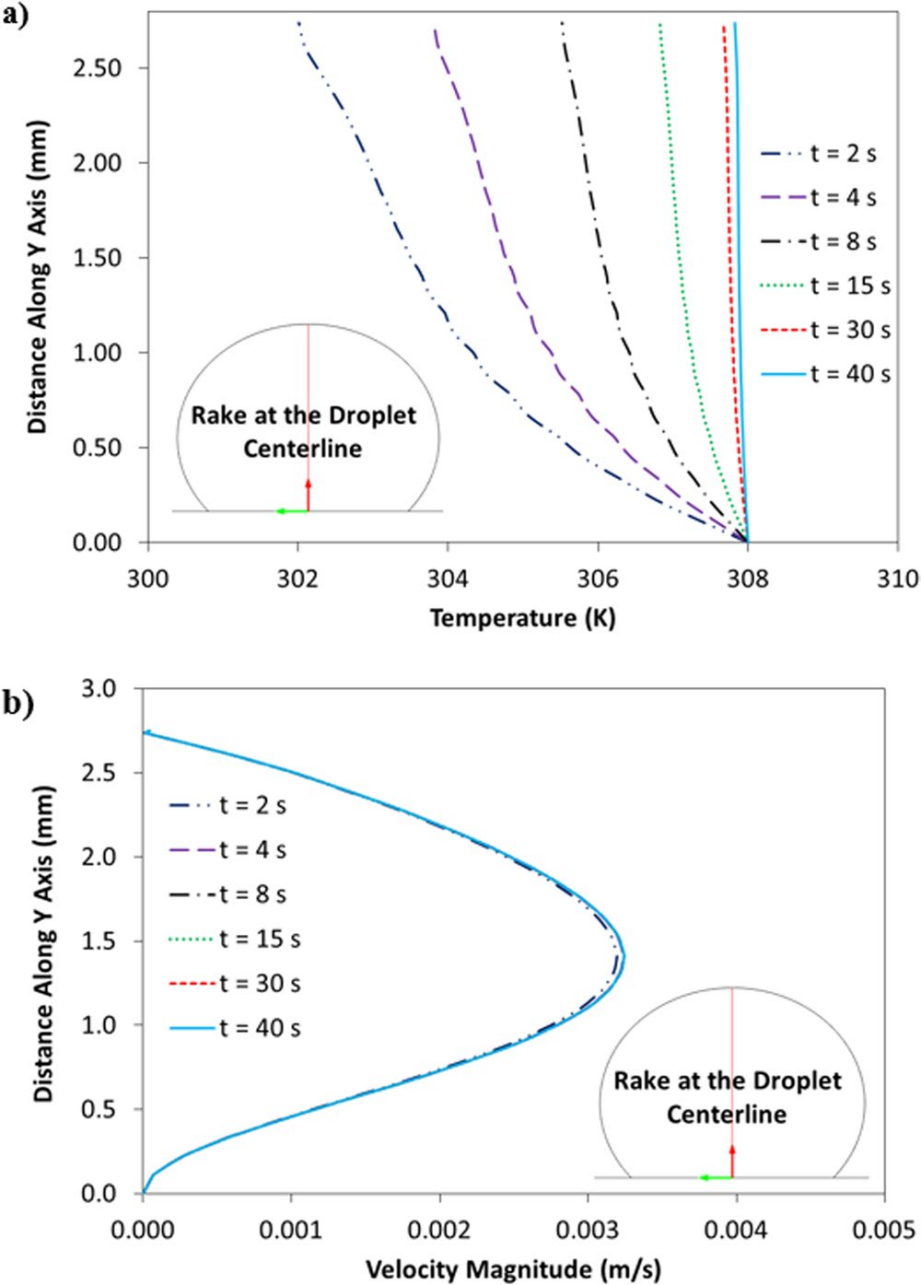
Temperature and velocity variation along the vertical distance in the droplet: (**a**) temperature variation for various times, and (**b**) velocity variation for various times. Droplet volume is 25 μL on horizontal hydrophobic surface.

**Figure 15:**
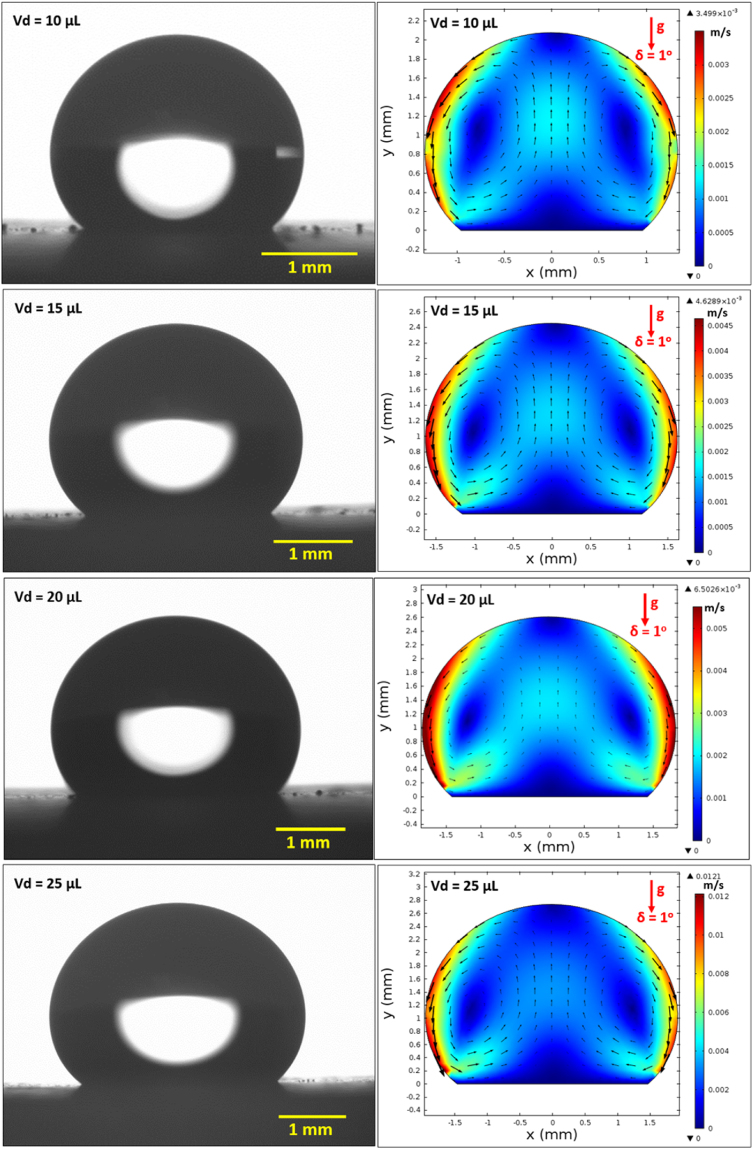
Optical images of droplets (on the left) and velocity contours (on the right) inside droplet for different volumes at 0° inclination angle.

**Figure 16:**
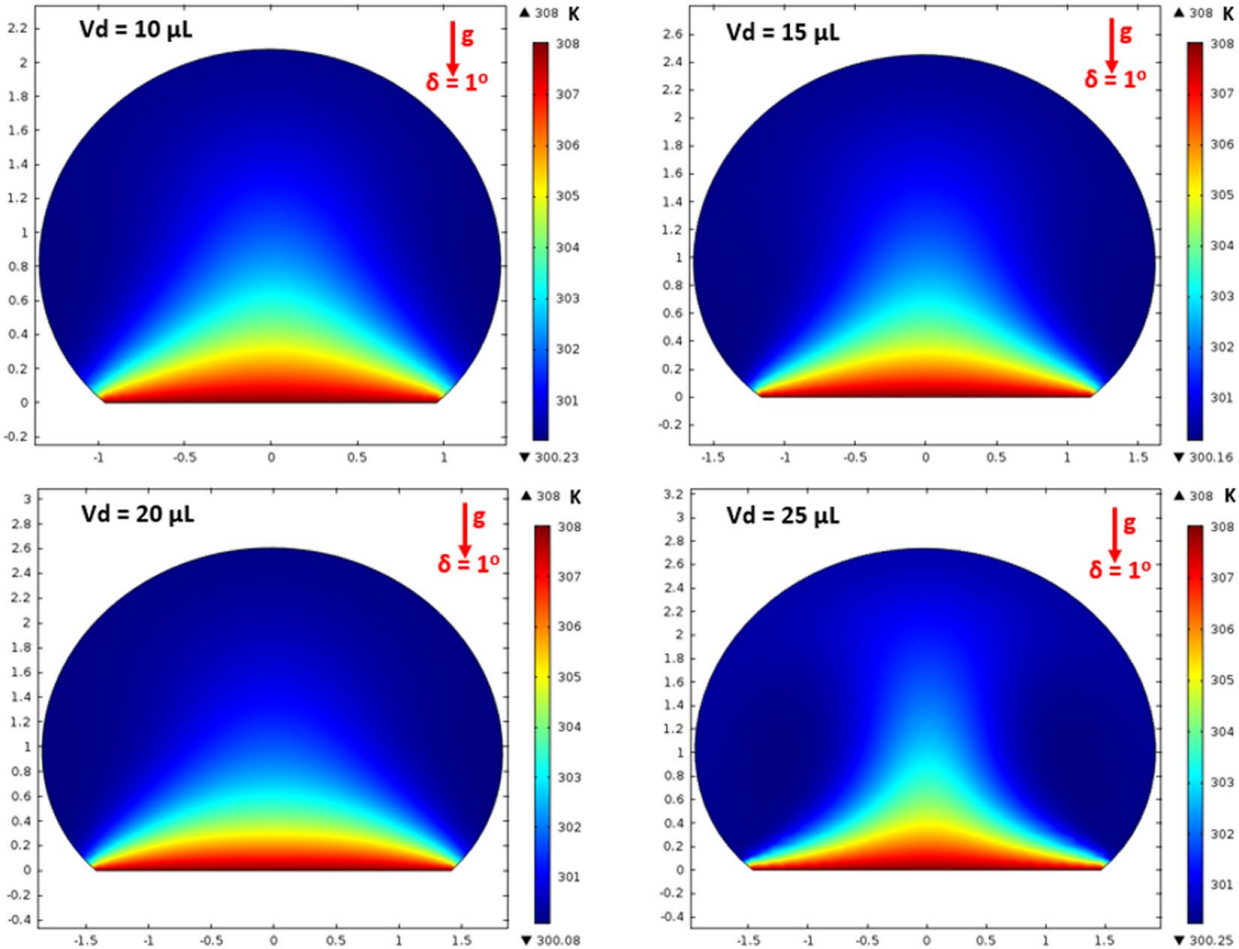
Temperature contours inside droplet for different volumes at 0° inclination angle.

**Figure 17:**
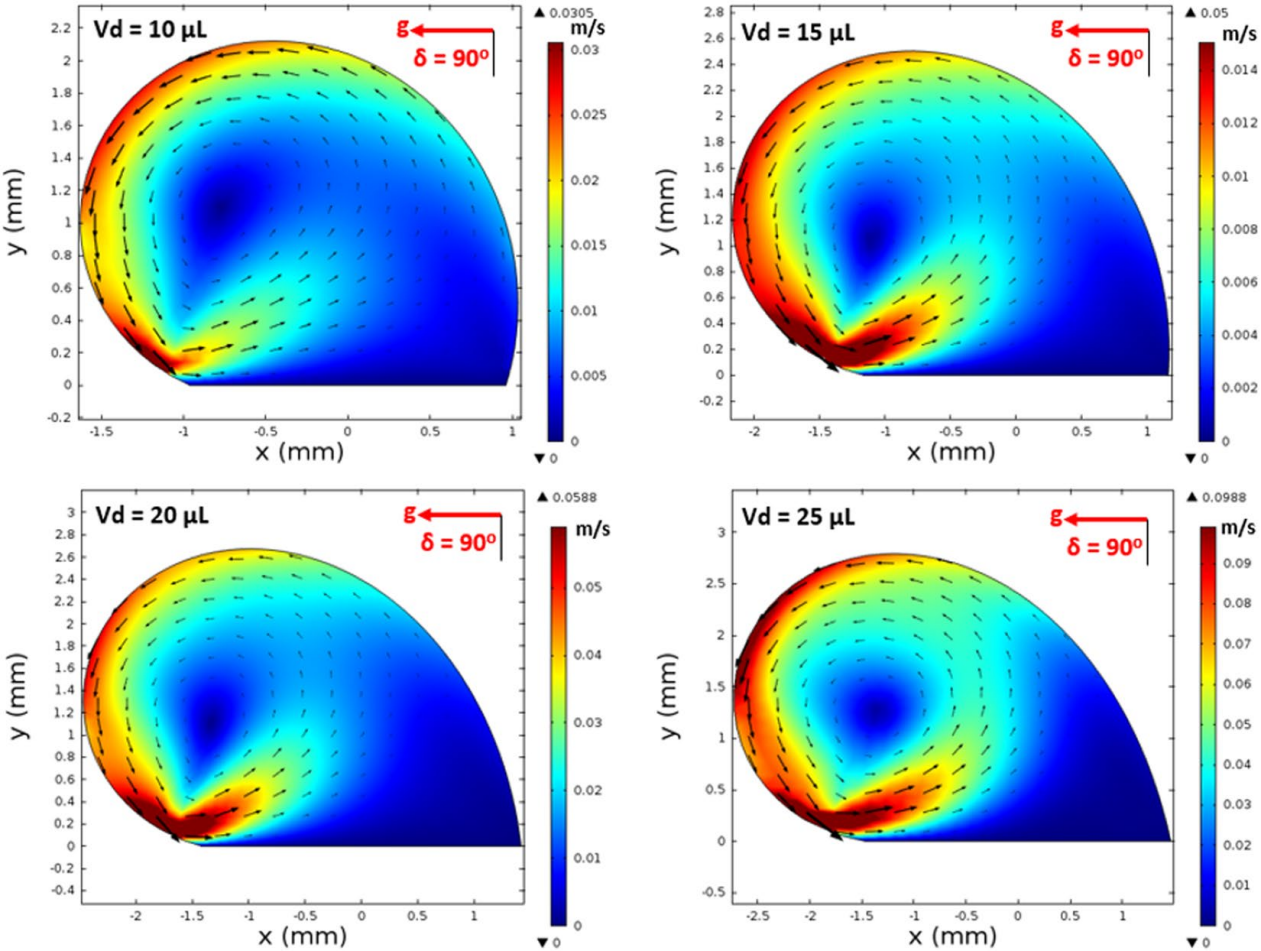
Velocity contours inside droplet for different volumes at 90° inclination angle.

**Figure 18:**
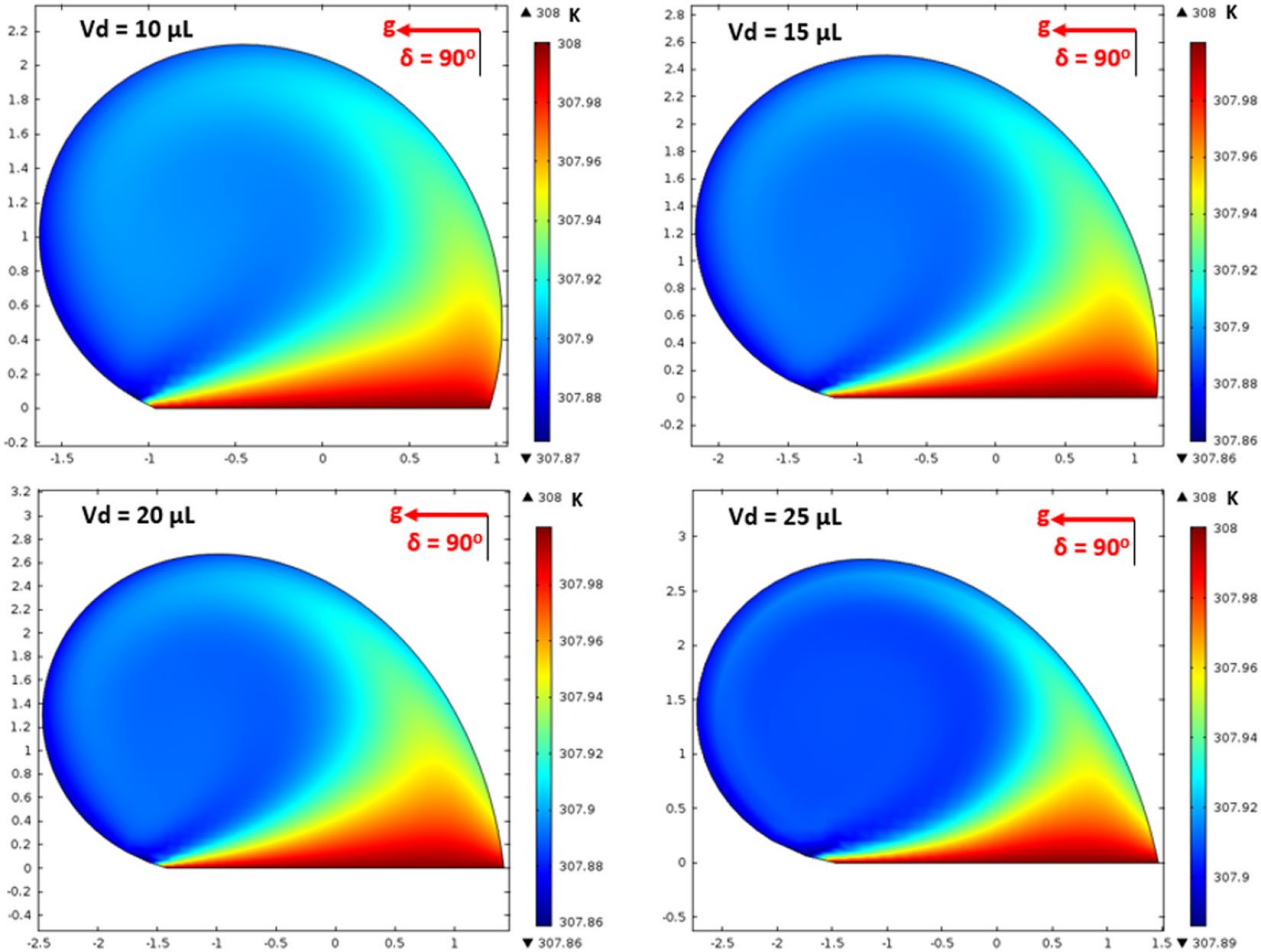
Temperature contours inside droplet for different volumes at 90° inclination angle.

**Figure 19:**
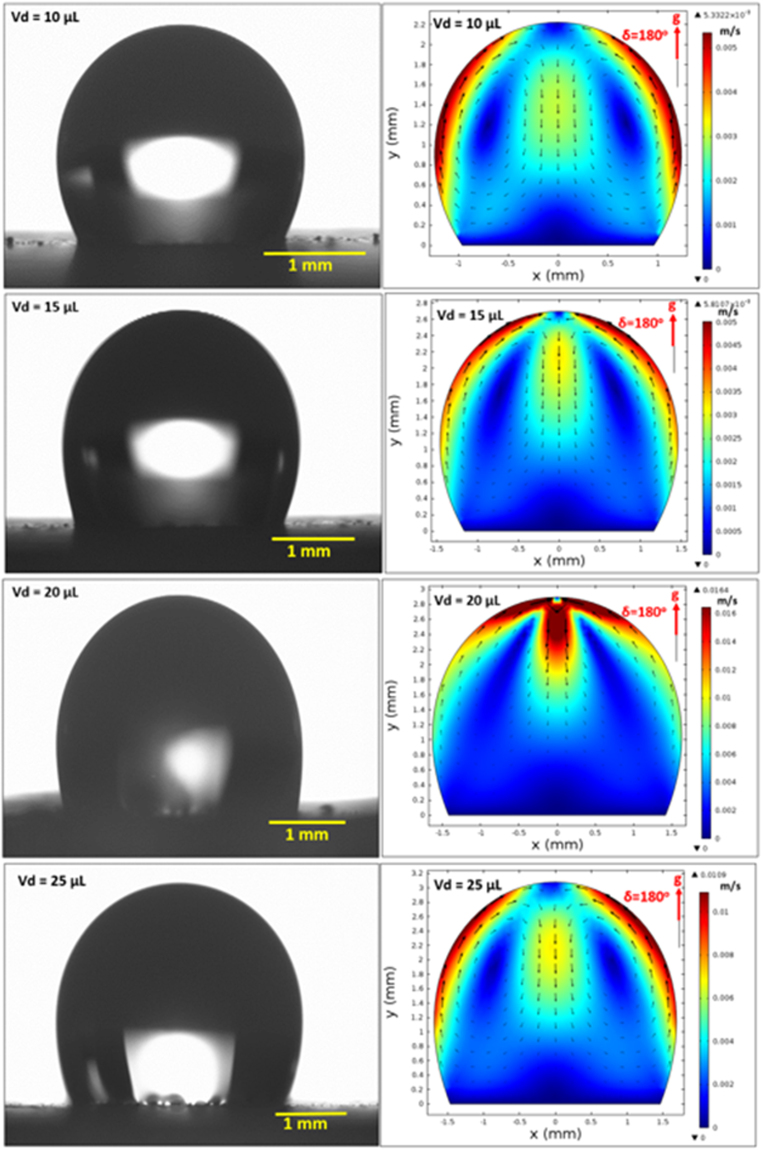
Optical images of droplets (on the left) and velocity contours (on the right) inside for different droplet volume at 180° inclination angle.

**Figure 20:**
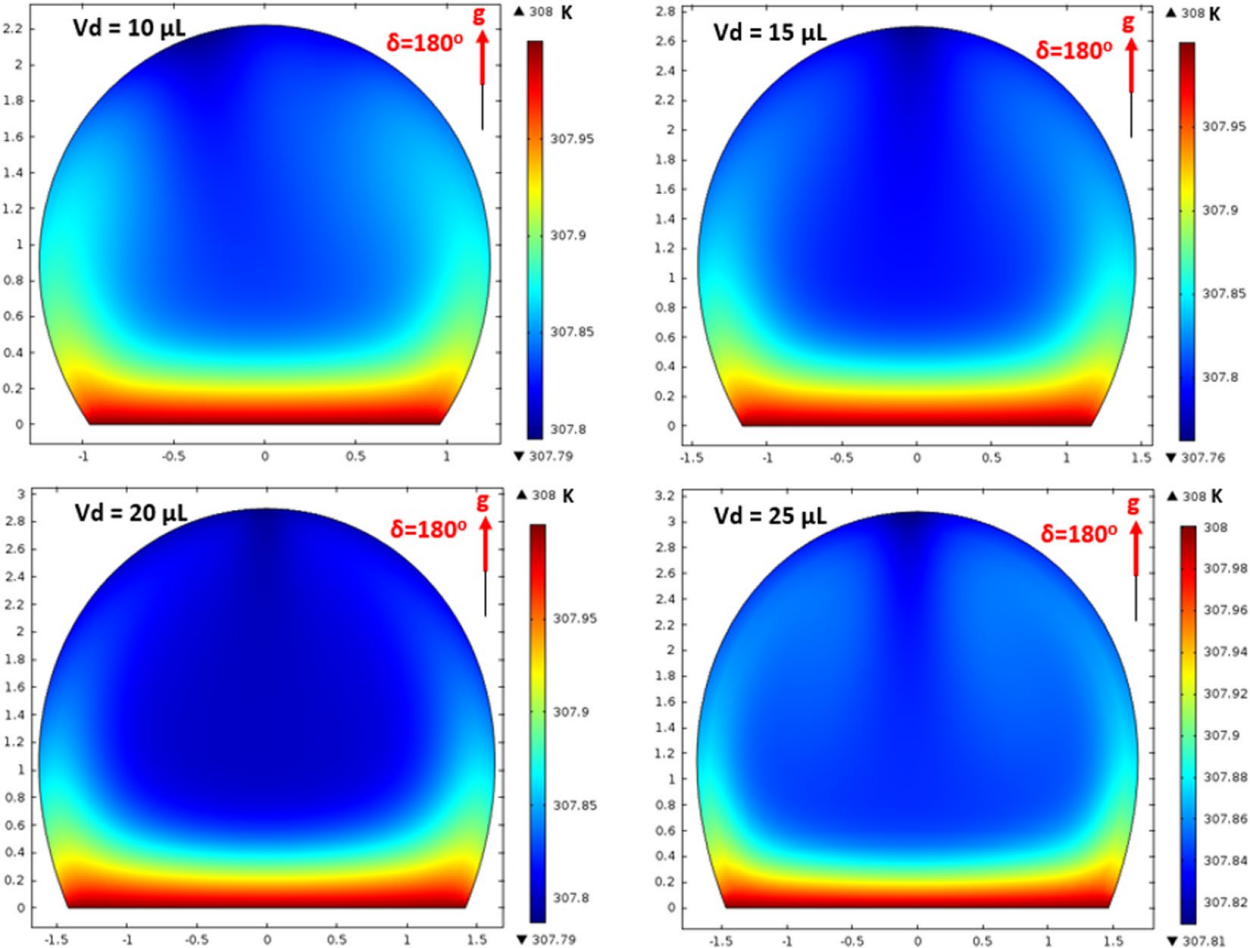
Temperature contours inside droplets for different volumes at 180° inclination angle.

On the other hand, in the analysis, internal fluidity of the droplet with heat transfer from the inclined hydrophobic surface is considered. In this case, the situation with a constant temperature heat source from the droplet bottom is considered to resemble the experimental conditions. Heat transfer modifies the flow field inside the droplet. The characteristic time scale associated with the thermal diffusion has the vital importance for the flow stability inside the droplet fluid. The Rayleigh number (Ra) mainly influences the flow stability in the buoyant convection^31^. The inclusion of the Marangoni effect in the simulations further influences the droplet instability. The Marangoni and the buoyant currents can result in a droplet rolling off from the hydrophobic surface during the heating period depending on the characteristic time for the stable flow. This situation possibly takes place for the small droplet diameters^31^. However, for a stable flow conditions, the ratio of square of the characteristic droplet diameter (*D*) over the thermal diffusivity of the droplet fluid (α_*T*_) should remain greater than unity ($\frac{{D}^{2}}{{\alpha }_{T}} > 1$)^31^. The characteristics time is estimated in the order of 30 s for the present case. Figure 14 shows the velocity and temperature variations along the vertical line inside the 25 μL droplet for various heating durations. The droplet contact angle is 135° and the hydrophobic surface is kept horizontal as shown in Fig. 14. Temperature (Fig. 14a) and velocity (Fig. 14b) varies significantly with time in the early heating period. As the time progresses variation of temperature along the vertical line collapses into a single curve. This behavior is also true for the velocity variation. The time for identical temperature profiles along the vertical line corresponds to 30 s, which satisfies the condition of$\,\frac{{D}^{2}}{{\alpha }_{T}} > 1$. Therefore, the time selected for presenting the flow and temperature fields satisfy the condition for the stable flow inside the droplet. Figure 15 shows the flow field inside the droplets on a horizontal hydrophobic surface together with the optical images of the droplets, prior to inclination, for various droplet volumes. Two rotating circulation cells with clock and counterclockwise wise rotations are formed inside the droplet. The circulation directions are towards the center of the droplet. The formation of counter rotating cells is associated with the Marangoni and buoyancy currents developed inside the droplet during the heating period. The velocity ratio of Marangoni flow over the natural convection is the same order of Marangoni over the Rayleigh number ($(\frac{\frac{\partial \gamma }{\partial T}{\rm{\Delta }}T}{{\alpha }_{t}\rho g{L}_{c}^{2}})$, where *γ* is the surface tension and $({L}_{c}=\frac{{V}_{d}}{\pi {R}^{2}})$ is the characteristic diameter)^31^; in which case, the velocity ratio remains greater than unity for a droplet characteristic diameter ≥2 × 10^−3^ m. Consequently, in the present case, the Marangoni current becomes larger than the buoyancy current while causing the formation of counter rotating circulation cells, which is consistent with the previous findings^16–18^. The heated fluid in the bottom region of the droplet is carried towards the droplet center by the Marangoni and the buoyancy currents. However, the size and direction of cell circulations can change with the droplet contact angle, which is particularly true for the heated and/or evaporating droplets on the hydrophilic surfaces with the droplet contact angle less than 90°^36–38^. Increasing the droplet size slightly alters the circulation cell center and velocity field inside the droplet. Figure 16 shows temperature contours inside the droplet for the horizontal hydrophobic surface and various droplet sizes. Since the heating takes place from the droplet bottom, heat transfer results in development of high temperature region in the close region of the droplet bottom. Hence, the convection current carries the heated fluid from the droplet bottom towards the fluid interior. In addition, flow circulation contributes to heat transfer towards the fluid interior and the attainment of high temperature region in the outer region of the circulation cell. This is more pronounced for the large droplet volumes. However, within the circulation cells, the heated fluid is not carried towards the circulation cell centers. Moreover, the heating is enhanced by both diffusion and convection. The large extension of the high temperature region takes place in the central region of the droplet bottom. This occurs because of: (i) heat transfer by heat diffusion in the lateral and normal directions, and (ii) convection heat transfer, i.e. heat carried by the convection current developed within the circulation cell exterior. Since the temperature gradient remains low in the lateral direction at the central location of the droplet bottom, heat diffusion takes place along the normal direction because of relatively lower temperature gradient along the normal direction as compared to that of the lateral direction. The heated fluid carried by the convection current slightly lowers the fluid temperature in the region of droplet edges. In addition, convection of cooling of the surface contributes to the attainment of low temperatures in the region in the edge regions. Figures 17 and 18 show velocity and temperature contours when the hydrophobic surface is inclined at 90° for various droplet volumes. Inclination of the hydrophobic surface alters the droplet shape significantly, which in turn modifies the flow field. The pressure distribution in the droplet changes because of the change of the gravity vector. In this case, a single circulation cell is developed inside the droplet, which extends almost covering the droplet interior. The circulation cell center and the velocity magnitude in the flow field changes with the droplet size. However, the maximum velocity inside the droplet attains lower values for 90° inclined surface than that corresponding to horizontally located droplet. This is more pronounced for the large volume droplets. Temperature field (Fig. 18) also differs from that is shown in Fig. 16. In this case, temperature increases along the outer region of the circulation cell. Consequently, thermal diffusion and temperature increase in the inner region of the circulation cell is not significant. However, thermal diffusion and the convection current are responsible for the extension of temperature field in the close region of the outer boundary of the circulation cell. When the droplet is inclined 180° from its horizontal position, the droplet shape changes significantly, this in turn modifies the pressure distribution inside the droplet. Consequently, the flow and temperature fields differ significantly from that of the horizontal droplet. This situation can be seen from Figs 19 and 20, in which velocity and temperature contours are shown for the 180° rotated droplet. The droplet shapes are also shown in Fig. 19 for comparison. Two counter rotating circulation cells are formed inside the droplet and the location of the cell center moves towards the droplet top edge unlike that is shown in Fig. 15. Consequently, change of the cell structure in the droplet modifies the heat transfer by convection and diffusion, and temperature distribution inside the droplet (Fig. 20). Since the magnitude of the flow velocity remains low, flow acceleration due to convection (*V*∂*V*/∂*s*), local (∂*V*/∂*t*), and radial (−*V*^2^/*R*, *R* being the droplet radius) effects remains low. The scale analysis for the acceleration depicts that the maximum radial accelerations is in the order of 10^−5^ m^2^/s, the convective acceleration is close to 0.005 m^2^/s and the local acceleration is about in the order of 10^−4^ m^2^/s. In this case, convection acceleration takes over the flow acceleration inside droplet during inclination of the hydrophobic surface. The shear stress and shear force due to the rate of fluid strain in the bottom region of the droplet are in the order of 3.3 × 10^−6^ N/m^2^ and 8 × 10^−12^ N, respectively, which is considerably smaller than that of the adhesion force (Fig. 12). Therefore, the influence of shear force developed in the droplet bottom region on the droplet pinning is negligibly small.

**Figure 21:**
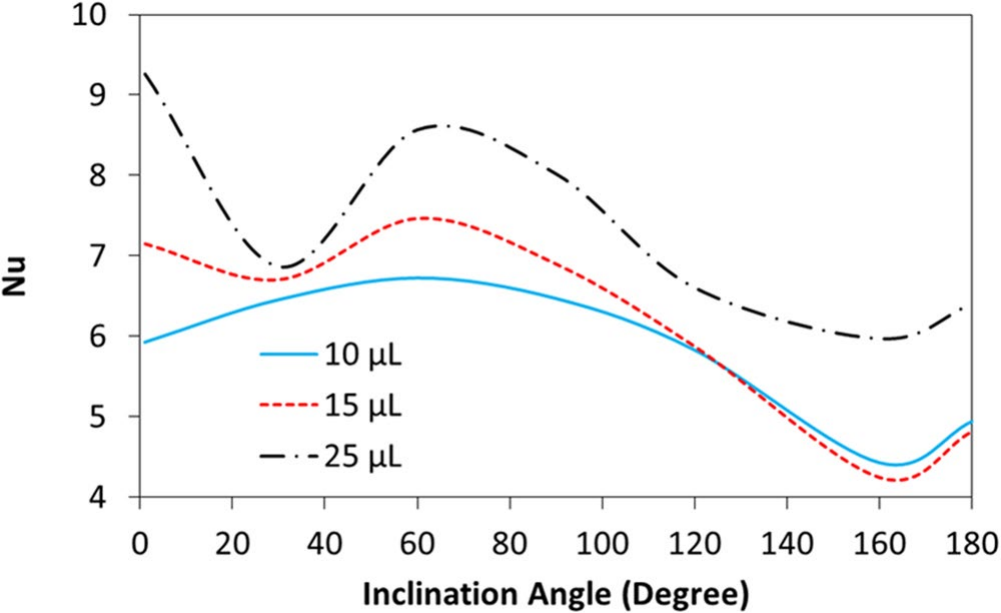
Nusselt number with inclination angle of hydrophobic surface for various droplet volumes.

Figure 21 shows the Nusselt number variation with the inclination angle of the droplet for various droplet sizes. It should be noted that the droplet pins during the inclination of the hydrophobic surface. The Nusselt number changes with the inclination angle of the hydrophobic surface; in which case, it reaches almost its peak value for the inclination angle of 65° and remains high for the inclination angle within range of 45° ≤ δ ≤ 90°. This behavior is attributed to the flow field developed inside the droplet, which influences the convection heat transfer from the droplet bottom towards the droplet interior. It should be noted that a single and large size circulation cell is developed inside droplet for the range of inclination angles in which the Nusselt number remains high. Consequently, heat carried by the convection current in the outer region of the circulation cell is mainly responsible for the heat transfer enhancement from the hydrophobic surface. In addition, heat diffusion in the region of the circulation cell outer boundary also contributes to this enhancement. As the inclination angle increases further, the Nusselt number reduces. The formation of two counter rotating circulation cells in the region away from the heated hydrophobic surface (Fig. 15) gives rise to reduced heat transfer rates from the surface, which is particularly true for the inclination angle of 180°. Increasing droplet volume improves the heat transfer and enhances the Nusselt number. This behavior is related to the fluid bulk temperature inside the droplet, which attains low values for the large volume droplets. Consequently, temperature difference between the hydrophobic surface and the droplet fluid becomes larger for large size droplet than that of the small size droplet while improving the Nusselt number.

## Conclusion

The pinning characteristics of a water droplet on an inclined hydrophobic surface are investigated and heat transfer from hydrophobic surface to the droplet is examined. Solution crystallization of a polycarbonate surface is carried out to texture the surface towards achieving the hydrophobic features. The resulting surface is characterized incorporating the analytical tools including 3-dimensional optical, scanning electron and atomic force microscopies, and X-ray diffraction. Water droplet contact angle and contact angle hysteresis are evaluated during the inclination of hydrophobic surface. The geometric features of the droplet for each inclination angle are recorded and the change of the droplet geometry due to bulging on the inclined hydrophobic surface is evaluated. The lateral and normal adhesion forces required for the pinning of the droplet on the inclined hydrophobic surface are formulated. Heat transfer from the inclined hydrophobic surface to the droplet is considered and the flow and temperature fields in the droplet are simulated in line with the experimental conditions. The predictions of flow field are validated incorporating the PIV data for flow velocity inside a droplet, which is pinned on the horizontal hydrophobic surface. It is found that the lateral adhesion force of the droplet overcomes the gravitational force in the direction of surface inclination, which is true for all the inclination angles and droplet volumes considered. The shear stress developed at the droplet bottom due to the rate of fluid strain is considerably smaller than that of the lateral adhesion force; hence, its contribution to the droplet pinning is negligibly small. The normal force component generated on the hydrophobic surface due to droplet surface tension is larger than that of the gravitational force for the surface inclination angles within the range of 70° ≤ δ ≤ 115°. As the inclination angle of the hydrophobic surface increases further, the normal component of the adhesion force remains less than the gravitational force resembling the component of the weight in the normal direction. Since the droplet pins on the inclined surface beyond 115°, the contribution of the Magdeburg like forces to the droplet pinning becomes critical. These forces are associated with the change of pressure in the air trapped within the texture gaps when the meniscus geometry of the droplet bottom changes during the inclination. Although some of the textures are fully connected and the air trap in these textures is exposed to atmospheric conditions, some of the textures are not connected and they appear as closed packed nature while causing the air pressure being different than the atmospheric pressure. The air trapped in these textures is responsible for the generation of the Magdeburg like forces at the interface of the droplet meniscus and the surface texture. Heating of the droplet causes formation of two contour rotating circulation cells inside the droplet for the surface inclination angles within the range of 0° < δ < 25° and 135° < δ < 180°. However, increasing the inclination angle further, a single circulation cell extends inside the droplet and occupies a large droplet volume, which is more pronounced for the inclination angles of 45° ≤ δ ≤ 135°. This behavior is attributed to the buoyancy and the Marangoni currents, which influence the flow field inside the droplet. The Nusselt number remains high for the inclination angles 45° ≤ δ ≤ 135°, which is attributed to the convection heat transfer inside the droplet; in which case, a single circulation cell is formed inside the droplet, and the enhancement of heat diffusion in the droplet fluid due to temperature gradient. The present study provides insight into the droplet adhesion on the inclined hydrophobic surface, and the forces acting on the lateral and the normal directions on the hydrophobic surface. A new concept of Magdeburg like forces contributing to the droplet adhesion on the extreme inclination angles of the surface is introduced. The details of the droplet heat transfer on the inclined hydrophobic surfaces are presented, which will be useful for fundamental underrating of droplet heating for practical applications.

## Electronic supplementary material

Supplementary Material S1
