# Supplementary material for: A Water Droplet Pinning and Heat Transfer Characteristics on an Inclined Hydrophobic Surface
Source: Sci Rep. 2018 Feb 15;8:3061. doi: 10.1038/s41598-018-21511-w (PMC5814416; doi:10.1038/s41598-018-21511-w)
Supplement: Supplementary file 1 — Supplementary Material S1 [file 41598_2018_21511_MOESM1_ESM.pdf]

Supplementary Information

A WATER DROPLET PINNING AND HEAT TRANSFER CHARACTERISTICS ON AN INCLINED HYDROPHOBIC SURFACE

Abdullah Al-Sharafi[1], Bekir Sami Yilbas [1, 2,] *, Haider Ali[1], N. AlAqeeli[1]

[1] Mechanical Engineering Department and Centre of Excellence in Renewable Energy, King Fahd University of Petroleum & Minerals, Dhahran, Saudi Arabia,

[2] Center of Research Excellence in Renewable Energy (CoRE-RE), King Fahd University of Petroleum and Minerals (KFUPM), Dhahran 31261, Saudi Arabia

*Corresponding author. Email: bsyilbas@kfupm.edu.sa; Phone: +966 3 860 4481

Analysis of Magdeburg Effects

Air is trapped in between the droplet meniscus when the water droplet is located on the textured hydrophobic surface. If some texture gaps (isolated gaps) are packed (not connected with air in the other texture gaps), pressure in the isolated gaps remains different than those of texture connected gaps. This, in turn, results in sealing of air trapped from atmospheric air because of droplet meniscus. As the droplet inclines, the meniscus arc changes slightly over the texture height giving rise change of the pressure in the trapped air while causing pressure force to be acting on the droplet meniscus. In case of expansion of the trapped air, due to slight volume change during the change of the geometric position of the droplet meniscus arc, a suction pressure is generated, which in turn results in Magdeburg like forces acting on the droplet meniscus. This contributes to the adhesion of the droplet on the surface. In the case of crystallized polycarbonate surface, texture composes of some closed packed gaps. Air volume inside the closed packed gap can be approximated by a half ellipsoid. The meniscus height across a single textured packed can be shown schematically in Fig. S1. Since micro-size spherules are formed on the crystallized polycarbonate surface, the spherules are presented as round textures in Figs S1a and S1b. The droplet meniscus height prior to bending can be formulated after incorporating the horizontal force balance. Consider Fig. S1a, the vertical force balance yields:

$$\rho g \pi a^2 h_d + \rho g \, \Delta\forall_d = F_\gamma \sin\theta \qquad (1)$$

where *mg* is the specific weight, h_d is the droplet height, F_γ ($F_\gamma = 2\pi a \gamma$) is the surface tension force, Δ_d is the volume of inflection. Consider $\Delta\forall_d$ is a half volume of an ellipsoid, then Eq. 1A becomes:

$$\rho g \pi a^2 h_d + \frac{1}{2}\rho g \frac{4\pi}{3} a^2 \chi = 2\pi a \gamma \sin\theta \qquad (2)$$

Since, $\sin\theta \approx \sqrt{\frac{\chi^2}{(a^2+\chi^2)}}$, and divide by $\rho g \pi a^2$:

$$\frac{2}{3}\chi - \frac{2\gamma\chi}{\rho g a}\sqrt{\frac{1}{(\chi^2 + a^2)}} + h_d = 0 \qquad (3)$$

The solution of eq. 3A yields the functional relation between the droplet meniscus height (χ) and the droplet height (h_d) in terms of fluid properties and lateral distance of the droplet meniscus across two consecutive texture pillars, where air is trapped.

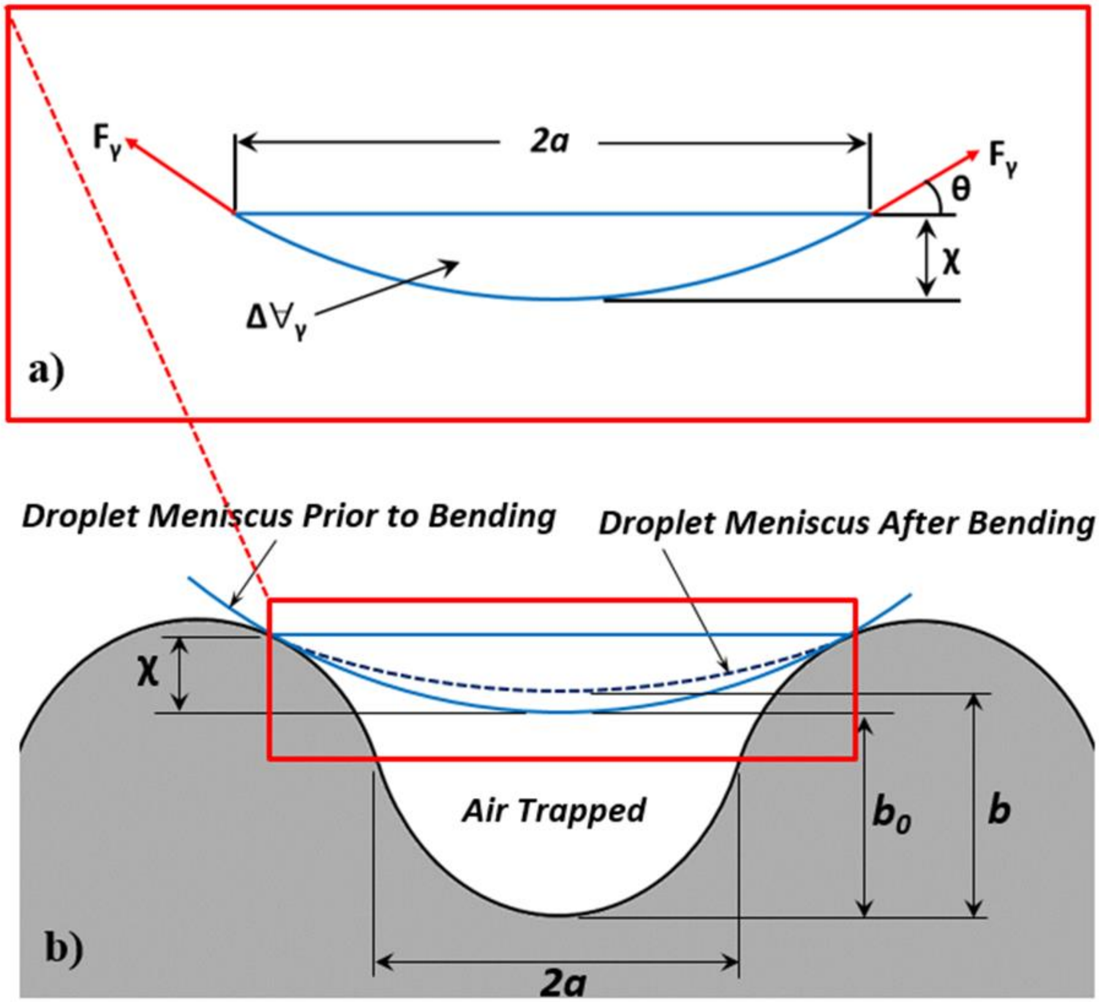

Figures S1. A schematic view of a closed packed texture gap: a) droplet meniscus across texture gap prior inclination of hydrophobic surface, and b) droplet meniscus across texture gap prior to and after inclination of hydrophobic surface.

The value of χ is in the order of 2.5 nm for 1 mm radius droplet and the spacing of closely spaced two spherules of 8 μm apart in the lateral direction, which is the same as 2*a* in Fig. S1a.

Consider the droplet meniscus prior and after inclination (Fig. S1b), the air trapped volume in a single gap can be formulated after approximating it to a half of an ellipsoid. After considering air is an ideal gas, the pressure drop in the air trapped during the geometric change of droplet meniscus, due to hydrophobic surface inclination, can be formulated as follows: Consider the equation of state for air:

$$P = \rho RT \tag{4}$$

where *P* is the pressure, ρ is the density, *R* is the gas constant for air, *T* is the temperature within the closed packed gap.

$$P = RT\frac{m}{\forall} \tag{5}$$

where $\forall$ is the air volume, and *m* is the air mass within the closed packed gap. In the differential form:

$$dP = -RTm\frac{d\forall}{\forall^2} \quad \text{`} \tag{6}$$

or

$$\Delta P = -RTm\int_{\forall_1}^{\forall_2}\frac{d\forall}{\forall^2} \tag{7}$$

Now, consider the half of an ellipsoid resembling air trapped volume, differential form of this volume yields:

$$d\forall = \frac{4}{3}a^2 db \tag{8}$$

Combining Eqs. 7A and 8A leads to:

$$\Delta P = -RTm\int \frac{\frac{4}{6}\pi a^2 db}{\left(\frac{4}{6}\pi a^2 b\right)^2} \tag{9}$$

or

$$\Delta P = \frac{6RTm}{\pi a^2}\left[\frac{1}{b} - \frac{1}{b_0}\right] \tag{10}$$

The pressure force generated in single packed texture gap is:

$$F = \pi a^2 \Delta P \tag{11}$$

The number of closed packed gaps on the crystallized polycarbonate surface is to be incorporated finding the the total pressure force acting on the droplet meniscus. Therefore, the approximate total pressure force is:

$$F_T = n\pi a^2 \Delta P \tag{12}$$

where *n* is the number of packed texture gaps on the crystallized polycarbonate surface. Combining Eqs. 10A and 12A results in the total pressure force, which becomes:

$$F_T = 6nRTm\left[\frac{1}{b} - \frac{1}{b_0}\right] \tag{13}$$

The area ratio of closed packed gaps sites over the total are of the crystallized polycarbonate surface is assessed using the texture height landscape image. In an averaged, the area ratio is estimated as in the order of 17%.

The pressure force (Magdeburg force) variation with the change of the height of the droplet meniscus within the air trapped in the closed packed is shown in Fig. S2. The pressure force increases significantly with small change of the droplet meniscus height in the closed packed texture gap. It should be noted that the area ratio estimated from the texture height landscape for the closed packed texture gaps is incorporated in Fig. S2. In addition, the average closed packed gap height is estimated as 3.4 μm from AFM data.

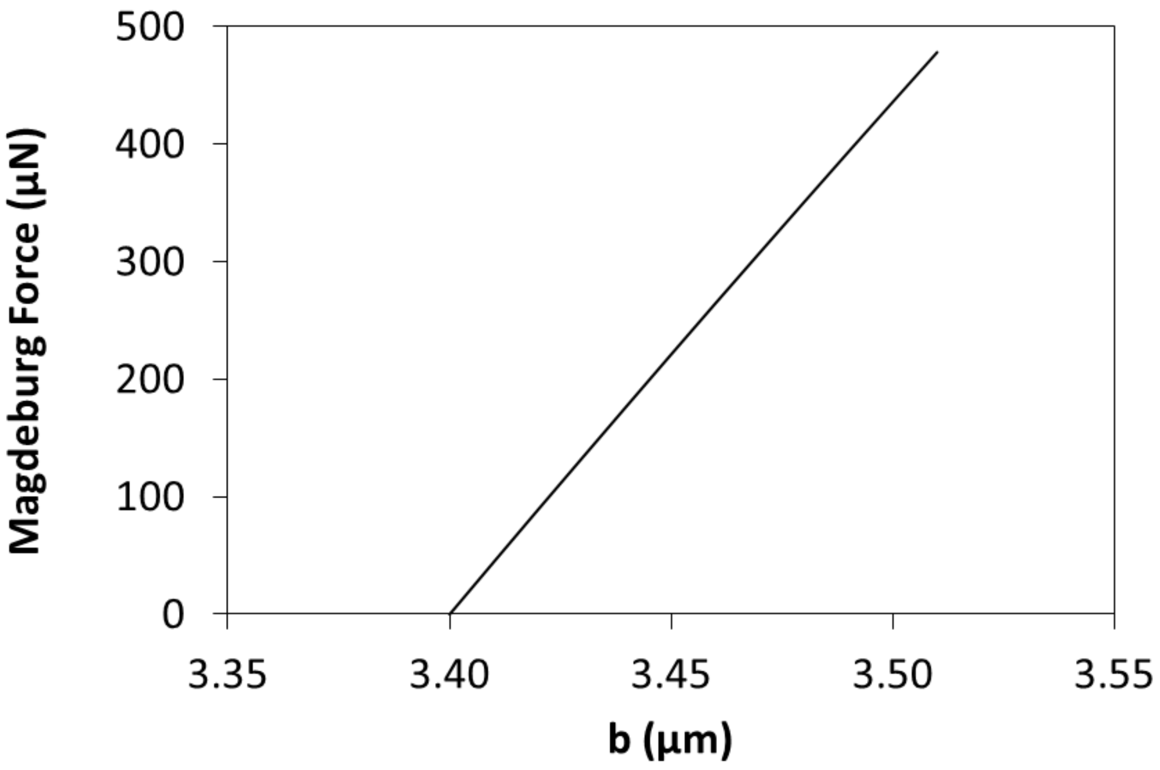

Figure S2. Magdeburg force with the height between droplet meniscus and the gap bottom after inclination of the surface.
